# Opportunistic CNS Viral Infections in Immunocompromised Patients

**DOI:** 10.3390/v18070800

**Published:** 2026-07-20

**Authors:** Adriana A. M. Giuliani, Nancy Law

**Affiliations:** 1 Division of Infectious Diseases and Global Public Health, Department of Medicine, University of California, La Jolla, CA 92093, USA; nalaw@health.ucsd.edu; 2 Division of Infectious Diseases, Rady Children’s Hospital, San Diego, CA 92123, USA

**Keywords:** viral encephalitis, opportunistic infection, CNS virus, herpes simplex encephalitis, JC virus encephalitis, measles encephalitis

## Abstract

Immunocompromised patients face increased morbidity and mortality from central nervous system (CNS) opportunistic viral infections, with risk level driven by specific immune defects. While our diagnostic capacity expands, immunocompromised patients may present atypically or with mild symptoms, delaying or complicating diagnosis. Treatment options are often limited, although novel agents show promise. This review discusses why immunocompromised patients carry higher immunologic risk and how certain viral classes display neurotropism to invade the CNS. We outline the utility and limitations of current and emerging diagnostic strategies. We discuss specific opportunistic viral pathogens, denoting specific epidemiology or pathogenesis, clinical presentation, diagnostic findings, and treatment and prevention methods. While there still remain knowledge and treatment gaps, advancements in diagnostics and understanding of pathogens have begun to improve outcomes.

## 1. Introduction

Opportunistic viral pathogens exploit weakened immune systems, more facilely invading new hosts or reactivating to cause disease. Immunocompromised patients, including those with primary or secondary immunodeficiencies or receiving immunomodulatory therapies, are particularly susceptible to severe and atypical opportunistic infections. Viruses with neurotropism, such as those belonging to the Herpesviridae family, may cause central nervous system (CNS) infection. While some may present with classic features of meningitis or encephalitis, immunocompromised patients are more likely to present in an atypical or subclinical fashion, making diagnosis more challenging. With the growing sensitivity of molecular testing, viruses may be identified in the CNS without clear significance or pathogenicity. Treatment is often limited in terms of effective antivirals and ultimately may depend on immune reconstitution, an impossible or difficult proposition conditional on the patient’s underlying diagnosis. In this review, we discuss the clinical presentation, diagnostic challenges, and current treatment approaches for key neurotropic viral CNS pathogens in immunocompromised patients, while also highlighting gaps in knowledge and areas for future investigation.

## 2. Immune Mechanisms and Pathophysiology

The blood-brain barrier (BBB) restricts passage of immune cells into the CNS [Klein]. Sparse antigen-presenting cells such as dendritic cells and macrophages may also limit immune response [1]. However, local T cells and B cells from the meningeal dura mater can protect the CNS from within, with constitutively secreted antibodies helping suppress and prevent infections [2]. Tissue resident memory T cells are also maintained in infected tissue, including the CNS, at areas of latent viral infection as continuous immune surveillance [3].

Microglia, astrocytes, and neurons all contribute to the native antiviral response in the CNS. Microglia and astrocytes contribute to type I IFN response, with microglia playing more of a responsive than productive role compared to astrocytes [4]. Astrocytes are the major IFN-β producers in certain viral infections such as VZV [4]. Microglia also act as antigen-presenting cells primarily to CD8+ T cells, while neurons have limited antigen-presenting capabilities [5]. Neurons display a lower-level baseline IFN response, with some heterogeneity; they favor autophagy as a defense mechanism to limit viral replication without triggering apoptosis [4].

Different immunocompromised populations face distinct CNS viral infection risks based on their specific immune defects. Response to and clearance of viral infections is primarily via cellular immune mechanisms enacted by CD4+ and CD8+ T cells, with the exception of enteroviral infections, which require intact humoral immunity driven by B cells [6]. 

### 2.1. Cellular Immunity

CD4+ T cells are critical to viral infection control both via direct cytolytic and noncytolytic mechanisms as well as the expansion, differentiation, and sustenance of the CD8+ T cell population [7,8]. CD4+ T cells limit polyomavirus infection in the kidney, a major viral reservoir, and regulate the emergence of polyomavirus variants, which can have increased neurovirulence [9]. They also modulate infection by VZV and CMV, promoting latency in neurons, while CD8+ T cells control HSV-1 infection primarily through noncytolytic mechanisms [7,10]. Profound CD4+ T cell depletion has been found to increase the severity of measles virus CNS infection in mouse models, as compared to CD8+ T cell or B cell deficiencies [11].

Defects in T cell function, particularly CD4+ T cells, can therefore lead to opportunistic infection by a wide breadth of viruses, including the Herpesviridae family, Polyomaviridae family, and measles virus. This introduces risk to those with primary T cell immunodeficiency, such as severe combined immunodeficiency (SCID) and secondary immunodeficiency, including persons living with HIV (PLWH), with risk and prognosis stratified by CD4+ count [12]. It may also affect patients exposed to T cell-depleting therapies such as those with hematopoietic stem cell transplant (HCT), solid organ transplant (SOT), graft-versus-host disease (GvHD), or hematologic malignancy. One study of HCT patients indicated a post-transplant risk of 1.2% for viral encephalitis, particularly after exposure to conditioning medications including alemtuzumab and OKT-3, with systematic review indicating increased mortality up to 37% compared to healthy populations [13,14]. Finally, certain immunomodulating therapies such as natalizumab, used in the treatment of multiple sclerosis and Crohn’s disease, can trigger progressive multifocal leukoencephalopathy (PML) caused by the JC virus [15].

### 2.2. Humoral Immunity

B cells classically produce antibodies but are also involved in T cell expansion and differentiation. B cell deficiency can increase risk of enteroviral infection, as antibody production is critical to enterovirus control [6]. Patients with agammaglobulinemia are known to present with persistent enteroviral meningoencephalitis [6].

See Table 1 for a summary of immunodeficiencies and associated infection risk.

### 2.3. Neurotropism and Portals of Entry

**Figure 1:**
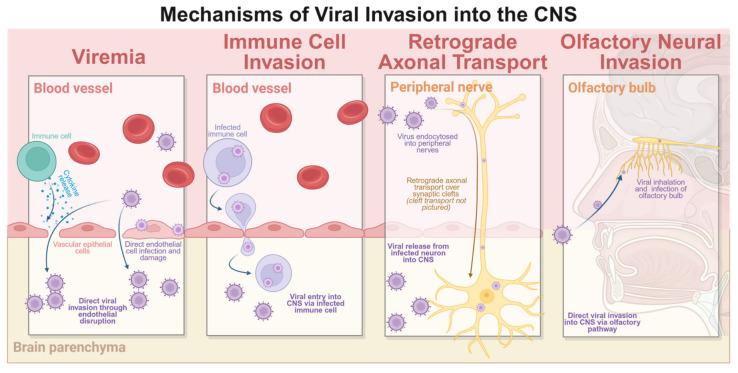
Depiction of Various Mechanisms of Viral Invasion into the CNS. Created in BioRender. Giuliani, A. (2026) https://BioRender.com/znudvil, accessed on 16 July 2026.

There are two primary mechanisms of CNS entry by neurotropic viruses: hematogenous spread and retrograde axonal transport [22,23]. While the BBB serves as a defense against infection, immunocompromised patients are more vulnerable, with the integrity of the BBB disrupted via direct and indirect endothelial damage, inflammatory cytokine release, and immune cell invasion [22,24]. Hematogenous spread may occur simply via viremia but is exemplified by immune cell infiltration, enabling Trojan horse mechanisms with which method viruses may penetrate into the CNS [22]. Viruses that target neurons enter by travel along peripheral nerves or crossing the BBB [22]. Certain viruses, such as HSV, VZV, and HHV-6 may also access the CNS through the olfactory pathway [22,25,26]. Different mechanisms of entry are illustrated in Figure 1.

## 3. Diagnostics

Cerebral spinal fluid (CSF) analysis remains the cornerstone of the diagnosis of viral encephalitis, bolstered or supplanted by other and emerging methods, including neuroimaging, serology, and brain biopsy.

**Figure 2:**
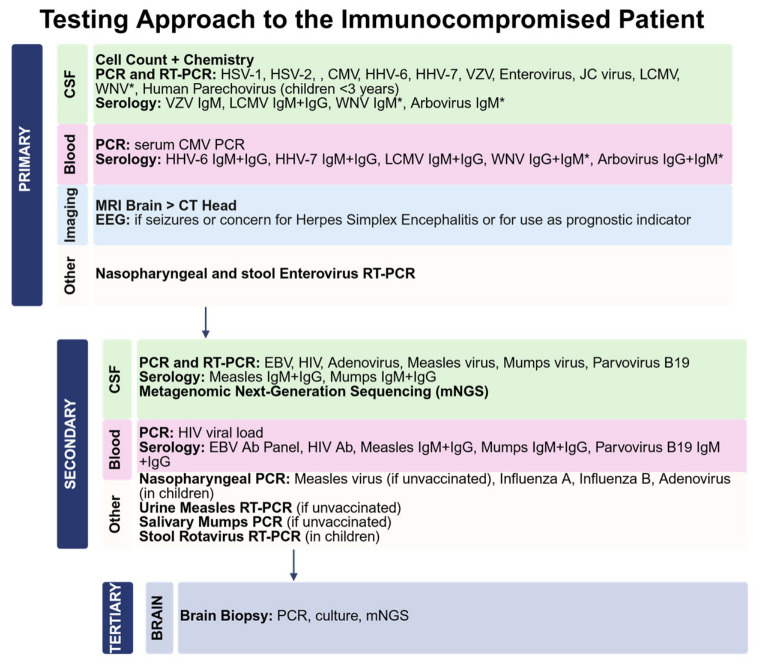
Testing Approach to Viral CNS Infection in the Immunocompromised Patient. “*” = based on season and geography. Adapted from Tyler et al. Created in BioRender. Giuliani, A. (2026) https://BioRender.com/s246lxb, accessed on 16 July 2026.

A tiered testing approach is recommended for both immunocompetent and immunocompromised patients, with the latter requiring expanded testing at earlier stages [27]. Initial testing rules out HSV-1, HSV-2, VZV, and enterovirus in all patients and should encompass CMV, HHV-6, HHV-7, JC virus, LCMV, and WNV in immunocompromised populations [27]. If negative or equivocal testing, next-generation sequencing should be pursued [27]. A tiered diagnostic approach for immunocompromised patients is detailed in Figure 2.

### 3.1. CSF Analysis and Molecular Diagnostics

As much CSF fluid as possible should be collected for analysis, with a minimum of 1 mL [28]. Larger volumes of 5–10 mL can increase culture sensitivity and are preferred for recovery of mycobacteria and fungi; however, small volumes are generally sufficient for viral testing [28]. The first tube should not be used for bacterial culture or molecular testing given a high risk of contamination [28].

Viral encephalitis may be marked by lymphocytic pleocytosis, elevated protein, and low-to-normal glucose; however, CSF counts may be normal in some patients, particularly among the immunocompromised. CSF viral culture has relatively low sensitivity, and polymerase chain reaction (PCR) testing is preferred [28,29]. HSV is cultured from CSF in only 5% of cases, compared to nucleic-acid amplification testing (NAAT) sensitivity and specificity of >95% for HSV encephalitis [28,29]. For enterovirus encephalitis, culture sensitivity is better at 65–75% but still substantially inferior to NAAT (>95% sensitivity) [29]. Viral culture is also labor intensive and may have prolonged recovery time-up to 8 days for enterovirus, further limiting its clinical utility [29]. CSF PCR generally carries good sensitivity and specificity, although it is distinct for each virus. However, some pathogens, particularly arboviruses, may have cleared viremia prior to symptoms of neuroinvasive disease, although viremia may be prolonged in immunocompromised patients [30]. PCR panels enable rapid detection of multiple common CNS viruses.

### 3.2. Metagenomic Next-Generation Sequencing (mNGS)

Metagenomic next-generation sequencing (mNGS) has enabled the identification of a broader range of pathogens, detecting all nucleic acids in CSF or brain tissue samples [31]. The PDAID prospective multicenter study demonstrated that among 204 patients with meningitis/encephalitis, mNGS identified 22% of infections (13/58) missed by conventional testing, with seven of these results leading to treatment changes [31]. It has allowed for identification of rare pathogens, including parvovirus 4, hepatitis E virus, and astrovirus, in otherwise undiagnosed encephalitis [27]. However, false positives may also occur due to environmental contaminants, so care should be taken in appropriate interpretation of results [31]. High CSF cell counts > 200 cells/mL^3^ can limit mNGS sensitivity given an overwhelming host DNA background [31]. It is not considered first-line testing given the high cost, limited availability, and potentially long turnaround time [31].

### 3.3. Serology

Serologic testing remains essential for pathogens poorly detected by PCR. However, antibodies may not be present in the acute phase of infection, and interpretation may be complicated due to antibody persistence from prior exposures [29]. Immunocompromised patients are less likely to display robust antibody response [32]. Serology testing is the preferred testing modality for arboviruses, including West Nile Virus, given NAAT sensitivity of <60% in immunocompetent patients, but may be affected by cross-reactivity between serogroups, requiring virus-specific confirmatory neutralizing antibody testing [28]. CSF IgM and IgG testing for VZV, EBV, and measles may also be considered when suspicion remains high despite negative PCR [27,28].

### 3.4. Neuroimaging

Magnetic resonance imaging (MRI) is the preferred imaging modality over computed tomography (CT) scan given improved sensitivity and specificity [27]. Certain imaging patterns may support the diagnosis of a certain viral etiology, although they can be nonspecific. Negative imaging does not imply lack of disease [27]. 

### 3.5. Electroencephalography (EEG)

EEG serves multiple roles, providing diagnostic support, clinical seizure monitoring, and prognostication value. EEG abnormalities are detected in 85.8% of viral encephalitis cases as compared to abnormal CSF findings in 57.3% of cases [33]. HSV encephalitis is marked by characteristic periodic lateralized discharges, which support diagnosis if detected [34]. Findings in other types of viral encephalitis are generally nonspecific [34]. Electrographic seizures can be detected in about 40% of patients with encephalitis [35]. A normal EEG is a strong predictor of survival [34].

### 3.6. Brain Biopsy

Brain biopsy may become required when other testing remains inconclusive, with diagnostic yields of 88–90% for focal CNS lesions and mortality of only 2–3% [14]. Biopsy should be considered in the presence of focal CNS lesions if the other noninvasive testing is negative or discordant, there is failure to respond to empiric therapy, or rapidly progressive neurologic deterioration without clear diagnosis [36]. Brain tissue may be sent for PCR, culture, and mNGS.

### 3.7. Emerging Diagnostics

Cell-free DNA/RNA analysis from plasma may be a less invasive alternative to CSF sampling; however, sensitivity is dependent on viremia, which is rarely present [37]. Systemic viral epitope scanning (VirScan), a comprehensive analysis of antibodies against all known human viruses via Phage-Immunoprecipitation Sequencing (PhIP-Seq), is in development but is not yet commercially available [38]. It is limited by complex analysis and long turnaround times [38]. Rapid CRISPR-based nucleic acid diagnostic tools are being developed, being used in one instance to diagnose Japanese encephalitis virus infection [39,40]. Cytokine and chemokine profiles may help distinguish between infectious and non-infectious etiologies, although they are not virus specific [41].

Characteristic diagnostic findings of specific viruses are reviewed in Table 2.

## 4. Herpesviridae

### 4.1. Herpes Simplex Virus-1 (HSV-1) and Herpes Simplex Virus-2 (HSV-2)

#### 4.1.1. Pathogenesis and Epidemiology

HSV uses retrograde axonal transport to establish itself within the trigeminal ganglia [24]. CNS infection can occur post-primary infection or after reactivation from latency, further utilizing retrograde axonal transport to infect new portions of the CNS, with predilection towards the temporal lobes and orbitofrontal cortex [24]. HSV-1 reactivation from latency may be promoted by glucocorticoid receptor binding, with patients on steroids at increased risk [58]. About 64% and 13.2% of the global population under 50 years old are infected by HSV-1 and HSV-2, respectively [59]. A recent multicenter study identified HSV as the most common etiology of encephalitis in immunocompromised patients, accounting for 18% of cases [60]. HSV-1 is the most common driver of herpes simplex encephalitis (HSE) [23]. HSV-2 infection can be seen in immunocompromised patients and is associated with 10% of total HSE cases [23]. HSE is the most common cause of non-epidemic acute focal viral encephalitis [23].

#### 4.1.2. Clinical Presentation

Patients present acutely with fever, encephalopathy, focal neurological deficits, and gastrointestinal symptoms [27]. Symptoms can progress over hours or days [24]. Seizures are common with HSV-1 encephalitis, although seen less frequently in immunocompromised patients [43]. HSV-2 is more likely to present as recurrent aseptic meningitis and is less likely to have long-term neurologic sequelae [14,61]. Mortality is 70% if untreated and remains high at 28% if treated [23,44]. Up to 27% of survivors suffer neurologic sequelae [44].

#### 4.1.3. Diagnosis

CSF studies typically show lymphocytic pleocytosis with moderate protein elevation and normal glucose [28]. CSF HSV PCR has high sensitivity and specificity [28]. However, reports of false negative HSV PCRs in the first 72 h have led to the recommendation to repeat CSF PCR after 3–7 days if HSE is still suspected [28]. Xanthochromia or elevated RBC can be a late manifestation given the hemorrhagic nature of the infection [44].

Brain imaging is often abnormal within 48 h [43]. CT and MRI imaging can demonstrate asymmetric signal intensity in mesial temporal lobes and extratemporal regions [43]. Persistence of imaging abnormalities can distinguish HSE from HHV-6 encephalitis, the latter imaging changes resolving more quickly [43]. EEG classically detects periodic lateralized discharges in the frontotemporal regions [34].

#### 4.1.4. Treatment and Prevention

In a retrospective review, mortality due to HSE was six times higher in immunocompromised patients [61]. Each one-day delay of acyclovir is associated with worsening functional status [61]. Therapy should be started empirically if HSE is suspected. Treatment is with acyclovir for 14–21 days in immunocompromised patients [62]. Acyclovir resistance may occur in up to 14% of immunocompromised patients, for whom foscarnet can be considered [49]. Adjunctive corticosteroids for cerebral edema are sometimes used, with a recent randomized control trial of dexamethasone for four days showing a good safety profile, although no improvement in neurologic sequelae [63]. Treatment for HSV meningitis is only for 10–14 days, and oral stepdown is an option [12,23].

There is no approved vaccine. Antiviral prophylaxis with acyclovir or valacyclovir is well-established to prevent mucocutaneous HSV reactivation in immunocompromised patients and remains part of post-transplant regimens [64,65]. However, it has not been specifically studied to prevent HSE [64,65].

### 4.2. Varicella Zoster Virus (VZV)

#### 4.2.1. Pathogenesis and Epidemiology

Most individuals are exposed to VZV by 9 years of age, after which it remains latent in the dorsal root ganglia [66]. As with HSV, VZV encephalitis may occur with primary infection or after reactivation [67]. Immunocompromised patients accounted for 39% of VZV encephalitis cases in a Danish cohort study [68]. PLWH with a CD4+ count < 200 cells/µL or with immune reconstitution inflammatory syndrome (IRIS) are at highest risk of VZV-related complications, including dissemination to the CNS [12].

#### 4.2.2. Clinical Presentation

VZV encephalitis usually presents as fever and encephalopathy, with focal neurologic signs and seizures less common as compared to HSE [68]. Rash may or may not be present [69]. VZV may cause vasculopathy, especially in immunocompromised individuals [67]. Infection of cerebral arteries can cause transient ischemic attack, stroke, aneurysm, and dissection [67]. Immunocompromised patients carry worse outcomes (24% with poor outcomes vs. 11%) [70].

#### 4.2.3. Diagnosis

CSF shows lymphocytic pleocytosis with high protein and normal glucose levels [42]. CSF PCR is quite sensitive at 80–95% in the immunocompromised population [29].

MRI may show temporal lobe involvement similar to HSV; however, VZV has characteristic ischemic lesions at the grey-white matter junction given its propensity towards vasculopathy [43]. Multifocal infarcts in different vascular territories may be seen [43].

#### 4.2.4. Treatment and Prevention

Delayed initiation of antivirals leads to poorer prognosis [70]. Acyclovir is the initial treatment of choice for VZV encephalitis, usually given at a higher dose than for HSE (10–15 mg/kg vs. 10 mg/kg) given decreased sensitivity of VZV to the antiviral [44,67]. Some experts recommend transitioning to oral therapy after clinical improvement and defervescence [12]. Total duration is 7–10 days; however, it is often extended to 14–21 days based on expert opinion, especially in the context of vasculopathy [12,71]. Adjunctive corticosteroids can be considered if vasculopathy is present [44]. 

Prophylaxis strategies are dependent on the patient’s underlying risk factor [66]. CMV prophylaxis regimens in SOT recipients is often sufficient for prevention of VZV; data is insufficient to recommend long-term prophylaxis [72]. HCT recipients are recommended to receive prophylaxis with acyclovir or valacyclovir for one year after transplant, with extension based on institution and type of transplant [73]. The varicella vaccine carries an efficacy of 80–85%, although effectiveness may be lower in immunosuppressed patients [66]. The recombinant zoster vaccine shows efficacy of around 70% in preventing viral reactivation [74].

### 4.3. Epstein Barr Virus (EBV)

#### 4.3.1. Pathogenesis and Epidemiology

EBV establishes lifelong latency in B lymphocytes, affecting >90% of the adult population [45,48]. A retrospective cohort analysis of 364 patients with encephalitis in China identified EBV in PCR specimens of 23.6% of cases; however, only 10.7% were considered to have EBV-associated encephalitis [45].

#### 4.3.2. Clinical Presentation

The presentation of EBV CNS disease spans meningoencephalitis, acute disseminated encephalomyelitis, acute cerebellar ataxia, neuropathies, and behavioral changes [75,76]. Symptoms are usually non-specific, with fever, headache, and encephalopathy [48]. Mortality can be as high as 10%, but outcomes are generally favorable [45].

EBV encephalitis is distinct from posttransplant lymphoproliferative disorder (PTLD), a disease driven by EBV leading to massive proliferation of infected B cells and to severe infiltrative disease and poor outcomes [77]. EBV can also be associated with primary CNS lymphoma in patients with AIDS or a history of transplant [78]. PTLD and primary CNS lymphoma are beyond the scope of this review.

#### 4.3.3. Diagnosis

CSF typically demonstrates pleocytosis with elevated protein and low glucose levels [45]. EBV PCR positivity in CSF does not consistently correlate with EBV encephalitis; EBV is detected in up to 24% of patients with acute encephalitis of other causes, therefore presenting a challenge to appropriate interpretation [28,45,47]. Quantitative PCR can help distinguish EBV encephalitis (high EBV load with pleocytosis) from CNS lymphoma (high EBV load with low white cell count) and post-infectious encephalitis or asymptomatic reactivation (low EBV load with low white cell count) [79]. The presence of EBV IgM in the CSF can be consistent with CNS disease; however, antibodies can be detected due to CSF contamination post-lumbar puncture or transfer of antibodies across a non-intact BBB [28,48].

There is a propensity for meningeal involvement in EBV encephalitis; however, imaging may be normal [45].

#### 4.3.4. Treatment and Prevention

Treatment recommendations are based on case reports and small case series. Acyclovir is the most used antiviral, although ganciclovir has more in vitro activity and is often administered in conjunction with steroids, which complicates assessment of antiviral efficacy [80]. Duration is not defined [80].

Rituximab, a monoclonal antibody to CD20, can target B cells to reduce the reservoir of infected cells [75]. It has poor penetration into the CNS and has been delivered intrathecally for treatment of refractory PTLD or CNS lymphoma [75]. Intrathecal rituximab has also been used in at least one case of transplant-associated EBV encephalitis, with CSF PCR clearance after two administrations and improved imaging [75]. Adoptive transfer of T cells is available for treatment of PTLD but has not been used in cases of EBV encephalitis [81].

Antiviral prophylaxis in SOT recipients is under debate; however, it may reduce the incidence of PTLD and EBV viremia in high-risk serologically mismatched patients [82]. Pre-emptive weekly serum EBV PCR screening is used in the HCT population, with reduction of immunosuppression or rituximab treatment implemented at certain thresholds [83].

There is no vaccine currently available, although several are in development [84].

### 4.4. Cytomegalovirus (CMV)

#### 4.4.1. Pathogenesis and Epidemiology

CMV is endemic worldwide, and the majority of people are exposed in childhood, establishing early latency in neurons [16]. Infection in PLWH is associated with CD4+ < 50 cells/µL [16].

#### 4.4.2. Clinical Presentation

CMV carries a wide breadth of neurologic presentations, including encephalitis, meningitis, vasculitis, retinitis, cranial nerve palsies, and radiculopathy [42]. Those with chronic encephalitis may present with lethargy and confusion, with or without fever [42]. Ventriculoencephalitis may present more acutely, progressing over days, with focal signs such as cranial nerve palsies and with rapid progression to death [42]. Labs may be notable for hyponatremia related to adrenal involvement [16].

#### 4.4.3. Diagnosis

CSF studies may show lymphocytic pleocytosis with low-to-normal glucose and normal-to-elevated protein levels [16]. PCR has good sensitivity of 82–100% as measured in PLWH [28]. Fewer than 50% of patients have imaging findings of periventricular enhancement on MRI or CT [16].

CMV viremia is not diagnostic of organ disease, including neurologic involvement, and negative CMV serum or plasma PCR does not rule out the presence of CNS disease [12].

#### 4.4.4. Treatment and Prevention

Ganciclovir treatment is first-line, with foscarnet used in cases to limit leukopenia or if there is evidence of ganciclovir resistance [16]. Combination therapy can be considered in severe or refractory disease [85]. Cidofovir may be used as a second line [85]. Maribavir is not recommended for the treatment of encephalitis given poor CNS penetration [85]. Optimal duration is not established, but treatment is recommended for at least 21 days [12].

Given poor outcomes in PLWH, it is recommended to initiate therapy with both ganciclovir and foscarnet [12]. A minimum of 21 days is recommended [12]. A randomized placebo-controlled trial did not show benefit for valganciclovir prophylaxis in PLWH considered high risk with low CD4+ count [12]. The best method of prevention is maintaining normal CD4+ counts through antiretroviral therapy (ART) [12].

Intravenous immunoglobulin (IVIG) is not recommended as adjunctive outside of CMV pneumonia [86].

Both pre-emptive (PCR screening) and prophylactic (antiviral administration) approaches are used in preventing CMV disease in transplant patients [85]. Letermovir is indicated only as prophylaxis [85].

### 4.5. Human Herpes Virus-6 (HHV-6)

#### 4.5.1. Pathogenesis and Epidemiology

HHV-6 is a ubiquitous virus, infecting a majority of the population in early life and establishing latency [52]. It is the most commonly identified virus in post-HCT encephalitis with an earlier incidence—before 100 days post-transplantation—as compared to other viral etiologies [13,14]. The reactivation risk is 30–80% post-HCT [52].

Importantly, HHV-6 can chromosomally integrate into host DNA, including germline cells [52]. When vertically transmitted from parent to offspring, known as endogenous HHV-6 (eHHV-6), the offspring carries a copy of the HHV-6 genome in all cells [52]. eHHV-6, formerly known as ciHHV-6 or iciHHV-6, is present in about 1% of the population, with the risk doubling in allogeneic HCT recipients as either the host or donor may have eHHV-6 [52]. It is transcriptionally silent and rarely causes disease but is associated with increased risk of GvHD and CMV reactivation [52].

#### 4.5.2. Clinical Presentation

HHV-6 may present as confusion, anterograde amnesia, and cognitive decline [87]. Encephalitis may be preceded by syndrome of inappropriate antidiuretic hormone secretion (SIADH), manifested as hyponatremia [52]. Sequelae include memory deficits and persistent altered consciousness [52].

#### 4.5.3. Diagnosis

Initial CSF analysis may be normal and acellular in immunocompromised patients [53]. CSF PCR has high sensitivity > 95% [18]. The presence of iciHHV6 can complicate interpretations of positive PCR. iciHHV-6 should be considered in cases of persistent viremia despite antiviral therapy or high copy numbers > 5.5 log_10_ in whole blood [52].

Early imaging within 2–7 days of symptoms is frequently normal [43,53,88]. Immunocompromised patients may develop limbic signal intensity, especially of the mesial temporal lobes; however, the changes do not tend to persist beyond 30 days as in HSV [43,52].

#### 4.5.4. Treatment and Prevention

Empiric treatment with ganciclovir and/or foscarnet should be considered for symptomatic patients with high suspicion of HHV-6 encephalitis [14]. Foscarnet shows superior antiviral activity and CNS penetration in in vitro studies but is limited by nephrotoxicity; ganciclovir carries the risk of myelosuppression [52,87]. There is no clinical data to recommend one over the other. Combination therapy may decrease neurologic sequelae, although it does not carry a mortality benefit [87]. Cidofovir has limited CNS penetration but may be considered third-line [52]. Treatment is prolonged, lasting weeks to months [50]. Virus-specific T cell therapy has been used in certain cases, but outcomes are inconclusive [52].

The median time of developing viremia to encephalitis is 4–5 days, limiting the efficacy of serial monitoring [52]. PCR screening in blood is not recommended in the absence of symptoms [52]. There is no vaccine or effective mode of prophylaxis [14].

### 4.6. Human Herpes Virus-7 (HHV-7)

While HHV-7 is ubiquitous with a seroprevalence of >95% in the general population, it is a rare but increasingly recognized etiology of CNS disease, causing meningitis, myelitis, and encephalitis [54]. HHV-7 is lymphotropic, primarily affecting CD4+ T cells [54]. In transplant recipients, HHV-7 is commonly identified as reactivated in blood; however, symptomatic disease is infrequent [89,90]. In HHV-7 encephalitis, patients usually present with at least one symptom of fever or focal neurologic finding; seizure is uncommon [54]. Foscarnet has had better case outcomes than ganciclovir and is preferred based on limited clinical data [54].

### 4.7. Human Herpes Virus-8 (HHV-8)

Seroprevalence of HHV-8 varies geographically, from 3 to 7% in the United States to 30 to >90% in sub-Saharan Africa [12]. There is an increased risk of infection in PLWH and men who have sex with men (MSM), with seroprevalence in MSM with HIV in the United States higher at 30–68% [12]. Involvement of HHV-8 in the CNS is scarce; however, may be seen in disseminated disease phenotypes including Kaposi sarcoma (associated with AIDS), primary effusion lymphoma, and multicentric Castleman disease [12]. Presentations are usually oncogenic, with exceedingly rare cases of encephalitis [55]. The primary intervention for disseminated HHV-8 disease is immune reconstitution, with adjunctive immunologic treatment or chemotherapy playing a role for the specific disease phenotype [12]. Antivirals such as foscarnet and ganciclovir have been utilized in encephalitis, having shown in vitro activity, however without proven clinical efficacy [55,90].

## 5. Polyomaviridae

### 5.1. JC Virus

#### 5.1.1. Pathogenesis and Epidemiology

There is 50–90% seroprevalence of JC virus among the general population [16]. It remains latent in kidneys, bone marrow, and lymphoid tissue [16]. CD4+ T cell dysfunction allows for viral escape from reservoirs and the development of neurovirulent variants [4]. Before the use of ART, PML occurred in 3–7% of patients with AIDS and was almost always fatal [12]. CD4+ T cell thresholds less than 200 cells/µL in PLWH increase the incidence of PML, although infection can also be seen with higher CD4+ counts [12]. Infection may be unmasked by IRIS in PLWH initiating ART therapy. Certain immunomodulating agents, including natalizumab (anti-α4 integrin medication), are strongly associated with PML [15].

#### 5.1.2. Clinical Presentation

Progressive multifocal leukoencephalopathy (PML) is a rare but dangerous complication of JC virus reactivation, causing demyelination via lysis of oligodendrocytes [15]. Common findings include focal deficits such as visual and speech defects, weakness, and loss of coordination [16,55]. Headache, fever, and seizures are less common [16,55]. Symptoms generally present over weeks to months but may present acutely with neurologic deficits [16]. If unable to reverse immunosuppression, the disease is usually fatal within a few months [15]. Many survivors suffer long-term neurologic sequelae [91].

#### 5.1.3. Diagnosis

Established criteria determine the certainty of PML diagnosis based on clinical, imaging, and CSF PCR findings, dividing likelihood into definite, probably, possible, and not PML [55]. CT imaging usually demonstrates hypodense lesions in affected white matter due to demyelination [55]. MRI is more sensitive and shows hyperintense lesions in the impacted areas [55]. Lesions are usually multifocal, although PML can also present as a solitary lesion and usually affect the subcortical white matter, cerebellum, and brainstem [16,55]. Unequivocal diagnosis of PML requires compatible clinical features, imaging findings, and positive CSF PCR; however, even with just the PCR, diagnosis remains possible or probable [55].

#### 5.1.4. Treatment and Prevention

Immune restoration is the backbone of treatment [16]. No antivirals have proven efficacy, although various agents have been attempted, with some studies aborted due to lack of benefit or high toxicity [53]. Strategies to achieve immune restoration include optimization of ART in PLWH, discontinuation of immunosuppressive therapy, and plasma exchange in natalizumab-associated PML [92]. Novel immunotherapeutic approaches, including allogeneic virus-specific T cell therapy and immune checkpoint inhibitors (pembrolizumab, nivolumab, atezolizumab), show promise [53].

The use of steroids in PML-IRIS is controversial, given the immunosuppressant effect may limit effective treatment of underlying HIV or disrupt other treatment plans [18]. A retrospective review demonstrated improved neurologic recovery with earlier initiation and longer duration of corticosteroids; another review failed to detect statistical significance but did note substantial clinical improvement in select steroid-exposed cases [93,94]. Guidelines recommend steroid initiation in case of severe clinical presentation or evidence of severe cerebral edema or mass effect, with dosing and duration tailored to the patient [12].

Rising antibody levels can precede PML in patients taking natalizumab, so JC virus antibody screening helps identify patients at higher risk [53].

### 5.2. BK Virus

BK virus is a ubiquitous virus that establishes early latency in uroepithelial cells and lymphocytes [49]. Small case numbers of BK virus meningoencephalitis have been reported in transplant recipients and patients with AIDS [49]. Immune reconstitution remains the cornerstone of treatment; as with JC virus, no antivirals have proven efficacy [95]. Cidofovir and adjunctive IVIG have been tried in clinical cases; however, they have variable effectiveness and poor outcomes [57,58].

Monthly screening for BK viremia is recommended until at least two years post-renal transplant [96]. Prospective screening of asymptomatic HCT recipients may identify patients at risk for kidney disease, CNS outcomes not being specifically studied [97].

Table 3 summarizes treatment strategies for Herpesviridae and Polyomaviridae CNS infections. Table 4 compares different antiviral medications and their utility in CNS infections.

## 6. Other and Emerging Pathogens

### 6.1. Measles

#### 6.1.1. Pathogenesis and Epidemiology

Measles is a human-only disease with high infectivity [113]. Acute encephalitis is reflective of direct viral invasion into the brain [114]. The cause of demyelination in post-infectious encephalitis is unknown but thought to be related to molecular mimicry [115]. Persistence of the measles virus in the brain, resulting in neurologic conditions months to years after primary infection, is posited to be from the fusion of infected and uninfected neurons, allowing for ongoing replication [114,115].

#### 6.1.2. Clinical Presentation

Measles encephalitis includes four overlapping presentations: (1) acute/primary, (2) post-infectious, (3) measles inclusion body encephalitis (MIBE), and (4) subacute sclerosing panencephalitis (SSPE), which are contrasted in Table 5. Acute encephalitis is due to direct viral invasion into the brain and presents as encephalopathy within a week of acute infection, intersecting with classic prodromal symptoms, which may be absent in immunocompromised patients [114]. Mortality and morbidity rates are relatively high at 30–40% [113]. Post-infectious encephalitis appears 2–30 days after acute infection [115]. MIBE is more likely to affect immunocompromised hosts, presenting up to one year after initial infection with rapid onset of encephalopathy and seizures and quick progression to death [113,115]. 75% of patients die within weeks of onset, and the rest are left with neurologic disability [113]. SSPE presents several years after infection, more common with young age at infection; however, symptoms may appear as an adult [114]. Patients tend to experience slow progression of cognitive impairment, seizures, and motor dysfunction until death in 2–3 years [114].

#### 6.1.3. Diagnosis

CSF parameters vary depending on the presentation, as reviewed in Table 4. CSF PCR is very sensitive [44]. CSF may show lymphocytic pleocytosis and elevated protein and glucose in acute and post-infectious encephalitis [114]. Testing can also include IgG and IgM of serum and PCR of throat, nasopharyngeal, or urine samples in early infection [44]. SSPE is diagnosed using Dyken’s Criteria, requiring two major and one minor criterion [116]. Major criteria include anti-measles Ab ≥ 1:4 in CSF or ≥1:256 in serum or compatible clinical history [116]. Minor criteria include characteristic EEG findings, elevated CSF globulin level > 20% of total CSF protein, characteristic histopathology on brain biopsy, and wild-type measles virus identified on molecular diagnostic testing [116].

Imaging is variable, depending on the type of presentation and stage of disease. Patients often have cerebral edema in acute encephalitis [114]. Post-infectious encephalitis, related to demyelination, may have multifocal asymmetric hyperintense lesions in the white matter on MRI [114]. SSPE is often normal early but may later demonstrate cerebral edema, atrophy, and/or multifocal lesions [114].

#### 6.1.4. Treatment and Prevention

High-dose vitamin A supplementation for adults and children is recommended by the World Health Organization given worsened outcomes in pediatric patients with vitamin A deficiency [117,118]. Treatment of post-infectious encephalitis may involve IVIG and/or plasmapheresis, and prognosis is generally favorable [113]. Remdesivir, inosine pranobex, ribavirin, IFNα, and IFNβ have been used for severe measles or SSPE but with unclear efficacy [113,114,119,120,121]. Monoclonal antibodies are emerging, with fusion protein-targeted antibodies promising to disrupt measles neural persistence [122].

Declining vaccination rates have led to a recent resurgence of the virus in North and South America, despite the possibility of eradication for a human-only disease [113,123]. Given high infectivity rates, 95% vaccination coverage is required to stop endemic transmission [123]. The live-attenuated vaccine is not safe for administration in certain immunocompromised populations, who are eligible for immunoglobulin prophylaxis if exposed to active virus [123].

**Table 5:** Comparison of Measles Encephalitis Types.

	Acute/Primary Encephalitis	Post-Infectious Encephalitis	MIBE	SSPE
Pathogenesis	Direct viral invasion into brain [113]	Autoimmune demyelination-possibly related to molecular mimicry [113]	Measles persistence is related to inability to clear virus [113]	Wild-type measles persistence (mutated noninfectious form in CNS) [113]
Timeline After Infection	Within 1 week [113]	Weeks to months [113]	Months to a year post-infection [113]	Years [113]
Presentation	Classic prodromal symptoms (may be absent in IC ^1^) + encephalopathy [114]	Encephalopathy, ataxia, motor and sensory deficits [113]	More common in IC ^1^ patients (ALL ^2^ highest risk; HIV, transplant, other cancer) [100] Seizures, encephalopathy, with rapid progression to death [113,124]	Gradual cognitive decline, seizures, and motor decline with inexorable progression to death [113]
Diagnostics	CSF PCR [44] CSF lymphocytic pleocytosis, elevated protein, elevated glucose [114] Serum IgG/IgM Throat/nasopharyngeal/urine PCR [44]	CSF lymphocytic pleocytosis, elevated protein [114]	Studies may be normal initially [113] CSF PCR usually negative 4× increase in CSF IgG/IgM [113] Definitive Dx ^3^: brain biopsy histopathology with +measles Ag or +measles RNA PCR [113,124]	Dyken’s Criteria: Major: elevated CSF or serum Ab, compatible clinical history Minor: characteristic EEG findings, elevated globulin level in CSF, brain biopsy histopathology, positive wild-type measles PCR [116]
Imaging	Cerebral edema [114]	MRI: multifocal asymmetric hyperintense lesions in the white matter [114]	Imaging may be normal initially [113] MRI: edema, atrophy, ventriculomegaly [113] EEG: non-specific changes [124]	MRI: normal early → cerebral edema, atrophy, multifocal lesions [113,114] EEG: periodic slow wave complexes during myoclonic spasm [113]
Treatment	High-dose Vitamin A supplementation if concern for deficiency [117] Ribavirin (in vitro data, no adequate trials) [119,120]	High-dose corticosteroids, IVIG, and/or plasmapheresis [113]	Symptomatic care [113]	Symptomatic care Compassionate use inosine pranobex, IFNα, IFNβ, remdesivir [113,121]

^1^ “IC”—immunocompromised, ^2^ “ALL”—acute lymphocytic leukemia, ^3^ “Dx”—diagnosis, “→”—leads to.

### 6.2. Other and Emerging Pathogens

See Table 6 and Table 7 for a review of opportunistic viral pathogens (Table 5) and arboviral (arthropod-borne) encephalitides (Table 6) not otherwise specified in the text.

## 7. Future Directions

Emerging immunosuppressants for oncologic, autoimmune, and rheumatologic diseases have broadened the population at risk for opportunistic viral infections. Pertinent screenings and a preventive approach should be determined based on their immune defect. While advanced diagnostic techniques such as VirScan and CRISPR-based tools will expand our capacity to detect microorganisms in the CSF, appropriate application of these tools should be considered prior to adoption. As measles incidence increases, it becomes more pertinent to delineate the overlapping presentations of encephalitis and define and develop treatment strategies.

Further studies are needed to determine optimal antiviral regimens and durations, as most recommendations are based on limited case experiences. Novel therapeutics, including antivirals, virus-specific T-cell therapies, and immune checkpoint inhibitors, may improve outcomes, particularly in diseases such as PML, retaining high mortality with prognosis incumbent on immunosuppression reduction. Limitations, including toxicity and poor antiviral penetration into the CNS, should be considered in drug development. Long-term neurocognitive outcomes and management still need to be assessed. Multiple vaccines are under development, which may change the landscape of risk, although safety, efficacy, and indication must be determined.

## 8. Conclusions

Opportunistic viruses exploit immune dysfunction to cause reactivation or severe primary infection in immunocompromised hosts, often involving the CNS through hematogenous or retrograde axonal spread. Neurologic infections in this population frequently present atypically or subclinically, complicating diagnosis. CSF PCR and neuroimaging remain the diagnostic mainstays, although findings may be normal, necessitating advanced testing such as mNGS or brain biopsy. While some CSF and imaging patterns may suggest specific etiologies, most findings are nonspecific. Mortality remains high, making early recognition, empiric therapy, and immune restoration critical. Emerging diagnostics may improve pathogen identification and differentiation of infectious from noninfectious encephalitis, though prospective studies are still needed to optimize antiviral treatment strategies.

## 9. Limitations

This review aims to cover the most common viral CNS pathogens in immunocompromised patients; however, it is not all-encompassing. The tables present common trends in presentation or diagnostic findings, but clinical cases, especially in those with immunodeficiencies, may present in an atypical fashion. Epidemiologic factors related to arboviruses (vectors, geography, risk factors, and preventive and vector control) are not reviewed. Arboviral presentations are not specific to the immunocompromised population. Treatment and diagnostic strategies are rapidly changing, and up-to-date information should always be pursued in the clinical care of a patient.

## Figures and Tables

**Table 1:** Select Immunodeficiencies and CNS Viral Infection Risk.

Immunodeficiency	Selected Indications	Mechanism	Associated Infection Risk
T Cell			
HIV/AIDS		Progressive CD4+ T cell depletion	Herpesviridae (CMV infection risk when CD4+ < 50 cells/µL) [16] Polyomaviridae (JC Virus infection risk when CD4+ < 200 cells/µL) [12] Measles virus [12]
Anti-Thymocyte Globulin (ATG)	Induction therapy for transplant [17]	Prolonged pan-T cell depletion [17]	Herpesviridae [17]
Calcineurin Inhibitors (Tacrolimus, Cyclosporine)	Post-transplant maintenance [17]	Suppress T cell activation by blocking IL-2 and IFN-γ production [17]	Herpesviridae [17]
Natalizumab	Multiple sclerosis, Crohn’s disease [18]	Monoclonal antibody to α4 integrin → prevents T cells from trafficking across the BBB ^1^ [18]	PML (JC Virus) [15]
B Cell			
X-Linked Agammaglobulinemia		Absence of functional B cells → agammaglobulinemia [19]	Enterovirus [6]
Common Variable Immunodeficiency (CVID)		Failure of B cell differentiation → decreased antibody secretion [20]	Enterovirus [20]
Anti-CD20 Inhibitors (Rituximab)	Hematologic malignancy, rheumatic diseases [18]	Monoclonal antibody to CD20+ B cells → circulating B cell depletion [18]	Enterovirus Parvovirus B19 PML (JC Virus)—correlation difficult to determine [18]
Combined			
Severe Combined Immunodeficiency (SCID)		Combined T and B cell immunodeficiency	Broad span of viral infections [21]
HCT Recipients		Neutropenia, T cell depletion, B cell depletion in early post-transplant period (especially with use of conditioning agents such as alemtuzumab and OKT-3) [13,14]	Broad span of viral infections

^1^ “BBB”—blood-brain barrier. “→”—leads to.

**Table 2:** Virus-Specific Diagnostic Features.

Virus	CSF WBC	CSF Protein	CSF Glucose	CSF PCR Sensitivity	Brain Imaging	Other
HSV-1/HSV-2	High (L ^1^) [42]	High [42]	Normal [28]	>95% [28]	Encephalitis: temporal lobe or inferior frontal lobe lesions [42] Meningitis: meningeal inflammation, frequently normal [23] Often abnormal early [43]	May have false negative PCR, repeat after 3–7 days if ongoing suspicion [28] Xanthrochromia or elevated RBC may be late finding [44] EEG with frontotemporal periodic lateralized discharges [34]
VZV	High (L ^1^) [42]	High [42]	Normal [29]	80–95% (IC ^4^) [29]	Variable, ischemia at grey-white matter junction [44]	
EBV	High (L ^1^) [45]	High [46]	Normal [46]	>95% (IC ^4^) [29]	Frequent meningeal involvement, may be normal [45]	Frequently reactivates (positive PCR) without disease- quantification can help distinguish [45,47] CSF IgM can help with diagnosis but can be false positive [28,48]
CMV	Normal or high (N ^2^ > L ^1^, M ^3^) [16,42]	Normal-high [16,42]	Low [42]	82–100% (PLWH ^5^) [28]	Periventricular lesions, ependymitis, ventriculitis (<50%) [42]	Presence of CMV in serum does not rule in or out CNS disease [12]
HHV-6	Normal or mildly high (L ^1^) [49]	Normal-high [50]	Normal [51]	>95% [29]	Limbic hyperintensity; often delayed [43,52]	May be acellular in early infection [52] Positive PCR complicated by eHHV-6 in normal population [52]
HHV-7	High (L ^1^) [50]	High [50]	Normal [53]	High (not otherwise specified) [53]	Non-specific, abnormalities in cortex, cerebellum, hippocampus; often delayed [50]	May represent asymptomatic reactivation [53]
HHV-8	Normal [54]	Normal [54]	Normal [54]	High (not otherwise specified) [54]	Non-specific (very rare) [12]	CSF parameters poorly defined given rarity
JC Virus (PML)	Normal, <20 [16,55]	Normal-high [16,55]	Normal [55]	>95% [55]	Hyperintense lesions at gray-white junction or brainstem white matter, usually multifocal [55]	Diagnostic criteria based on clinical findings, imaging, and CSF PCR [55]
BK Virus	Normal [56,57]	Normal [56]	Normal [56,57]	High (not otherwise specified) [56,57]	Variable, non-specific [56]	
Enterovirus	High (L ^1^/N ^2^) [42]	Normal-high [42]	Low-normal [42]	>95% [28]	Usually normal but EV71 may cause dorsal brainstem, cerebellar, and spinal cord lesions [44]	

^1^ “L”—lymphocytic, ^2^ “N”—neutrophilic, ^3^ “M”—monocytic, ^4^ “IC”—immunocompromised, ^5^ “PLWH”—persons living with HIV.

**Table 3:** Summary of Treatment and Prevention of Major CNS Viral Infections.

Virus	First-Line	Alternative/Adjunctive	Duration	Prevention
HSV-1/HSV-2	Encephalitis: IV Acyclovir 10 mg/kg Q8H [12] Meningitis: IV Acyclovir 10 mg/kg Q8H → PO Valacyclovir 1 g TID transition if clinically stable, afebrile [12]	Foscarnet (acyclovir-resistant) [12]	Encephalitis: 14–21 days [12] Meningitis: 7–10 days [12]	Oral Acyclovir or Valacyclovir in post-Tx ^1^? [64,65]
VZV	IV Acyclovir 10–15 mg/kg Q8H → transition to PO Valacyclovir 1 g TID once afebrile and clinical improvement if no visceral involvement [12]	Foscarnet (acyclovir-resistant) [12] Steroids in case of vasculopathy or severe disease [44]	7–10 days [12], ≥14 days per expert opinion [71]	PO Acyclovir or Valacyclovir in Tx ^1^ population [72,73] Varicella vaccine [66] Recombinant zoster vaccine [74]
EBV	IV Acyclovir or IV Ganciclovir 5 mg/kg Q12H → PO Valganciclovir 900 mg daily [80]	IT ^2^ Rituximab (single case report) [75] Steroids can be considered but low evidence [80] Virus-specific T cell therapy [81]	Not defined (days to weeks) [80]	No vaccine [84]
CMV	IV Ganciclovir 5 mg/kg Q12H and/or IV Foscarnet (60 mg/kg Q8H or 90 mg/kg Q12H) [12,85] Combination therapy is first-line in PLWH [12]	Cidofovir [85]	≥21 days [12]	PLWH: none, maintain CD4+ with ART [12] Tx ^1^ patients: pre-emptive (PCR screening) or prophylactic (antiviral) [85]
HHV-6	IV Ganciclovir 5 mg/kg BID and/or IV Foscarnet 90 mg/kg BID [52] → PO Valganciclovir 900 mg daily [14]	Cidofovir [52] Virus-specific T cell therapy [52]	Weeks to months [50]	No vaccine or prophylaxis [14]
HHV-7	Foscarnet (limited data) [54]	Ganciclovir (less effective per case data) [54]	Not established	
HHV-8	Reduction of immunosuppression [12]	Foscarnet + Ganciclovir [55,91]	14 days [55,90]	
JC Virus	Reduction of immunosuppression [16]	Virus-specific T cell therapy [53] Immune checkpoint inhibitors [53] PML-IRIS: consider adjunctive corticosteroids [18,93,94]	Prolonged [16]	Monitor antibody levels if on natalizumab [53]
BK Virus	Reduction of immunosuppression [97]	Cidofovir [57,58] Corticosteroids [57,58]		BK virus PCR screening [97]

^1^ “Tx”—transplant, ^2^ “IT”—intrathecal, “→”—followed by.

**Table 4:** Antiviral Medications and CNS Penetration.

Drug	Mechanism of Action	CSF: Plasma Ratio	Plasma Protein Binding Rate	Adverse Effects
Acyclovir	Purine nucleoside analog [98]	0.31 (uninflamed meninges) [99] ~0.50 [98]	9–33% [98]	Neurotoxicity (confusion, hallucinations, dysarthria), renal impairment (renal tubular damage), thrombotic thrombocytopenic purpura (TTP)/hemolytic uremic syndrome (HUS) in IC ^1^ [98]
Valacyclovir	Prodrug of Acyclovir [100]	0.19 (uninflamed meninges) [99] ~0.50 [100]	9–33% [100]	Neurotoxicity (confusion, hallucinations, dysarthria), AKI (renal tubular damage), thrombotic thrombocytopenic purpura (TTP)/hemolytic uremic syndrome (HUS) in IC ^1^ [100]
Ganciclovir	2′-deoxyguanosine, requires viral protein kinase pUL97 phosphorylation [101]	0.31–0.68 [101]	1–2% [101]	Fever, diarrhea, leukopenia, anemia, neurotoxicity (less common than Acyclovir) [101]
Valganciclovir	Prodrug of Ganciclovir [102]	Equivalent to Ganciclovir after conversion [102]	1–2% [102]	Fever, diarrhea, leukopenia, anemia, neurotoxicity (less common than Acyclovir) [102]
Foscarnet	Viral DNA polymerase inhibitor [103]	0.66–0.69 (steady state) [103]	14–17% [103]	Renal impairment, seizures (including status epilepticus), electrolyte abnormalities, QTc prolongation [103]
Cidofovir	Viral DNA polymerase inhibitor [104]	Undetectable in CSF [104]	6% [104]	Renal impairment, neutropenia [104]
Maribavir	CMV pUL97 kinase inhibitor [105]	Very low (not recommended for CNS disease) [105]	98% [105]	Dysguesia, nausea, vomiting [105]
Ribavirin	Nucleoside analog [106]	Very low if taken orally; intraventricular administration is required for CNS disease [107]	0% [106]	Teratogenic, hemolytic anemia [106]
Remdesivir	Nucleotide analog [108]	0.26 (single case report) [109]	88–93.6% [108]	Hypersensitivity reaction, transaminitis [108]
Oseltamivir	Neuraminidase inhibitor [110]	Very low (0.029–0.035) [111]	42% (pro-drug), 3% (active metabolite) [110]	Nausea, vomiting, headache [110]
Rituximab	Monoclonal antibody to CD20 antigen [112]	CSF penetration extremely poor; intrathecal administration required [76]	Not defined (monoclonal antibody) [112]	Severe mucocutaneous reaction, Hepatitis B reactivation [112]

^1^ “IC”—immunocompromised.

**Table 6:** Other and Emerging Pathogens.

Virus	Clinical Presentation	Diagnosis	Treatment
Adenovirus	Encephalitis, meningitis Mortality up to 80% in HCT patients [125]	CSF: counts normal, PCR often positive [125] MRI: non-specific [125]	Cidofovir (limited evidence) [125] Ribavirin (limited evidence) [125] Adoptive T cell therapy (emerging) [126]
Enterovirus	Common in B cell deficiencies [6] Encephalitis, meningitis, acute flaccid paralysis-dependent on strain [127]	CSF: lymphocytic pleocytosis in 80% [127], PCR >95% [29] but low sensitivity for EV-D68 [128] Imaging: usually normal but EV71 may cause dorsal brainstem, cerebellar, and spinal cord lesions [44]	IVIG in IC ^1^ patients or severe disease [129] Pocapavir (viral capsid inhibitor) in compassionate use [19]
Human T-cell Lymphotropic Virus-1 (HTLV-1)	HTLV-1 Associated Myelopathy (HAM): acute (over weeks), subacute, or chronic inflammatory disease of spinal cord [130] Encephalopathy [130]	CSF: CSF HTLV-1 proviral load > serum proviral load indicates higher risk of HAM [130] MRI: extensive transverse myelitis (subacute HAM), confluent frontoparietal white matter and corticospinal tract lesions (encephalopathy) [130]	Corticosteroids [130] IFNα (effective but limited use related to side effects) [131,132] Mogamulizumab (decreases viral load, unclear clinical efficacy) [133]
Influenza	Children more often than adults [134] Acute seizures, high fever, encephalopathy → coma [134] Mortality 27% [135]	CSF: normal-to-high WBC, normal-to-high protein [134] MRI: cerebral edema [134] EEG: normal or with diffuse slowing [134]	Neuraminidase inhibitors (oseltamivir, peramivir) Adjunctive methylprednisolone, IVIG, plasma exchange, tocilizumab [134,135]
Lymphocytic Choriomeningitis Virus (LCMV)	Fever, headache, vomiting → brief remittance → encephalitis, meningoencephalitis [136] May be donor-derived in SOT with high mortality [137]	CSF: PCR, IgG/IgM [137] Serum: acute/convalescent IgG/IgM [137]	Supportive care, reduction in immunosuppression Ribavirin (unclear efficacy) [137] Adjunctive corticosteroids, IVIG [137]
SARS-CoV-2	Impaired consciousness, aphasia, behavioral abnormalities [138]	CSF: PCR negative in majority of cases (more likely inflammatory than invasive), normal-to-high WBC, normal-to-high protein [138] MRI: variable but usually abnormal, involving temporal, medial temporal, and frontal regions [138] EEG: non-specific, diffuse slowing [138]	Supportive care, corticosteroids [138] Remdesivir for acute systemic infection (not specific to CNS disease)

^1^ “IC”—immunocompromised, “→”—leads to.

**Table 7:** Arboviral Pathogens.

Virus	Clinical Presentation	Diagnosis	Treatment
Alphavirus			
Chikungunya Virus	Neuroinvasive: meningoencephalitis, myelitis, myeloneuropathy [44,139] Several weeks after prodromal phase [139]	CSF: pleocytosis, elevated protein [139]	Supportive care [139] Vaccine available
Eastern Equine Encephalitis Virus	<5% neuroinvasive (RF ^2^: age > 50, age <15, IC ^1^) [140] Nonspecific febrile illness → headache, encephalopathy, focal neurologic deficits, seizures, coma [140] Mortality up to 75%, sequelae in 50% [140]	CSF: pleocytosis (N ^3^ → L ^4^), elevated protein, normal glucose; IgM (may be negative in IC ^1^); mNGS [30,140] MRI: focal abnormalities of basal ganglia, mesial temporal lobes, thalami, and brainstem [30,140] EEG: generalized slowing [140]	Supportive care (limited data for IVIG; steroids did not show survival benefit) [140] Human vaccines in pre-clinical studies; current vaccine in equines [140,141]
Western Equine Encephalitis Virus	Rare human cases, majority asymptomatic Mortality 10–15% [142]	CSF: IgM, PCR [142]	Supportive care [30] Vaccine in pre-clinical studies [141]
Venezuelan Equine Encephalitis Virus	Fever, pharyngitis, meningismus [143] Mortality low [141]	CSF: +/− pleocytosis (L ^4^), elevated protein, low glucose; PCR [143]	Supportive care Vaccine in pre-clinical studies [141]
Bunyavirus			
Jamestown Canyon Virus	Meningitis, meningoencephalitis More common in adults than children [144]	CSF: pleocytosis (L ^4^), elevated protein, normal glucose, IgM, PCR [144] MRI: non-specific hyperintensities [144] EEG: generalized slowing [144]	Supportive care [144]
La Crosse Virus	Encephalitis > meningitis [144] Fever, seizures (42–62%), focal neurologic deficit (16–25%) [145] More common in children than adults [144] Risk of herniation related to cerebral edema [145] Sequelae in 10–15% [23]	CSF: pleocytosis (may be hemorrhagic, mimic HSE ^5^) [145] MRI: cortical enhancement, generalized cerebral edema [145] EEG: diffuse slowing, discharges may mimic HSE ^5^ [145]	Supportive care [145]
Coltivirus			
Colorado Tick Fever Virus	Biphasic illness in 50% Children: rare meningoencephalitis, can be fatal Adults: prolonged weakness and fatigue [146]	CSF: IgM, RT-PCR (first two weeks) [147]	Supportive care [146]
Flavivirus			
Dengue Virus	50% of encephalitis without typical syndrome (myalgia, rash, bleeding) [148] Neuroinvasive: meningoencephalitis (0.5–6.0% cases), GBS ^6^, myositis [44,148]	CSF: pleocytosis (L ^4^), IgM (ratio > 1.5 of serum IgM), NS1 Ag (less sensitive than blood), PCR, mNGS [148] MRI: hyperintensities in basal ganglia, thalamus, cerebellum, cortical grey and white matter; micro-hemorrhages; “double doughnut” sign [148]	Supportive care, possible IVIG and corticosteroids [148] Direct-acting antivirals in early clinical trials [148] Vaccine available, efficacy to reduce severity [148]
Japanese Encephalitis Virus	Rapid fever, unconsciousness, meningismus [149] Seizures in 85% of children and 10% of adults, associated with poorer outcome [23] 30% mortality, 30–50% neurologic sequelae [23,150]	CSF: elevated opening pressure [149,151]; pleocytosis, elevated protein, normal glucose [149]; antigen (low sensitivity) [149]; IgM (high sensitivity) [149]; PCR [150] MRI: mixed-intensity or hypodense lesions of thalamus, basal ganglia, and midbrain [149]	Supportive care Vaccine available [23,149]
Powassan Virus	Febrile prodrome [152] Neuroinvasive: rhombencephalitis >> encephalitis, meningitis, meningoencephalitis, acute flaccid paralysis, opsoclonus-myoclonus syndrome [152] Mortality 10%, sequelae in >50% [152,153]	CSF: lymphocytic/mixed pleocytosis, elevated protein, normal glucose; IgM; RT-PCR MRI: hyperintensity in basal ganglia, thalami, brainstem, cerebellum, cortex EEG: generalized slowing [152]	Supportive care [152]
St. Louis Encephalitis Virus	Encephalitis, meningitis, anterior horn-cell paralysis [27] Neuro involvement more common in adults [44] Seizures associated with poorer outcome [154] Urinary symptoms (dysuria, pyuria) and SIADH [44] Mortality 3–30% [44]	CSF: elevated opening pressure [154]; pleocytosis (L ^4^), elevated protein, normal glucose; IgM; PCR; mNGS [154]	Supportive care [155]
Tick-Borne Encephalitis Virus	Meningitis (35–45%), meningoencephalitis (45–55%), meningoencephalomyelitis (10%) [156] Monophasic or biphasic course (flu-like symptoms → neurologic), can be acute or progressive [156] Neurologic sequelae in 40–50% (paralytic or post-encephalitic) [157]	CSF: pleocytosis (N ^3^ → L ^4^), IgM; RT-PCR (low sensitivity, viremia cleared by time of neurologic symptoms) [156,157] MRI: abnormalities of thalamus > cerebellum, basal ganglia, brainstem (low sensitivity); hyperintensity of anterior horns of cervical cord if myelitis or radiculitis [156] EEG: diffuse slowing, focal abnormalities [156]	Supportive care (prior IVIG use discontinued with concern for worsened course) [156] Multiple vaccines available [157]
West Nile Virus	<1% lead to neuroinvasive disease [158] Meningitis: headache, stiff neck, photophobia [158] Encephalitis: confusion, +/− tremors, myoclonus, ataxia in severe disease [158] Acute Flaccid Myelitis (AFM): acute asymmetric limb weakness within 24–48 h of disease, +/− cranial neuropathy → respiratory failure [118] Mortality 30–40% in IC ^1^ patients [158]	CSF: pleocytosis (L ^4^), elevated protein, normal glucose, PCR (more sensitive than Ab in IC ^1^), IgM [158] Serum: IgM [158] MRI: may be normal, outcomes worse if abnormalities present [158] - Encephalitis: hyperintensity in brainstem and deep gray matter [158] - AFM: hyperintensity in anterior horns or around conus medullaris and cauda equina [158] EEG: non-specific with generalized slowing, triphasic sharp waves [158]	Supportive care (attempted treatments underpowered or ineffective) [158]

^1^ “IC”—immunocompromised, ^2^ “RF”—risk factor, ^3^ “N”—neutrophilic, ^4^ “L”—lymphocytic, ^5^ “HSE”—herpes simplex encephalitis, ^6^ “GBS”—Guillain-Barré Syndrome, “→”—leads to.

## Data Availability

Data sharing is not applicable No new data were created or analyzed in this study.

## Abbreviations

The following abbreviations are used in this manuscript:

CNS	Central nervous system
HSV	Herpes simplex virus
VZV	Varicella zoster virus
EBV	Ebstein Barr virus
CMV	Cytomegalovirus
HHV	Human herpesvirus
PLWH	Persons living with HIV
HCT	Hematopoietic stem cell transplant
SOT	Solid organ transplant
GvHD	Graft-versus-host disease
PML	Progressive multifocal leukoencephalopathy
BBB	Blood-brain barrier
CSF	Cerebral spinal fluid
PCR	Polymerase chain reaction
NAAT	Nucleic-acid amplification testing
mNGS	Metagenomic next-generation sequencing
MRI	Magnetic resonance imaging
CT	Computed tomography
EEG	Electroencephalography
HSE	Herpes simplex encephalitis
PTLD	Posttransplant lymphoproliferative disorder
ART	Antiretroviral therapy
IRIS	Immune reconstitution inflammatory syndrome
IVIG	Intravenous immunoglobulin
IC	Immunocompromised
HTLV-1	Human T-cell lymphotropic virus-1
LCMV	Lymphocytic choriomeningitis virus

## References

[1] Klein R.S., Hunter C.A. (2017). Protective and Pathological Immunity during Central Nervous System Infections. Immunity.

[2] Cardani-Boulton A., Boylan B.T., Stetsenko V., Bergmann C.C. (2022). B Cells Going Viral in the CNS: Dynamics, Complexities, and Functions of B Cells Responding to Viral Encephalitis. Immunol. Rev..

[3] Gebhardt T., Palendira U., Tscharke D.C., Bedoui S. (2018). Tissue-resident Memory T Cells in Tissue Homeostasis, Persistent Infection, and Cancer Surveillance. Immunol. Rev..

[4] Telikani Z., Monson E.A., Hofer M.J., Helbig K.J. (2022). Antiviral Response within Different Cell Types of the CNS. Front. Immunol..

[5] Buckley M.W., McGavern D.B. (2022). Immune Dynamics in the CNS and Its Barriers during Homeostasis and Disease*. Immunol. Rev..

[6] Bearden D., Collett M., Quan P.L., Costa-Carvalho B.T., Sullivan K.E. (2016). Enteroviruses in X-Linked Agammaglobulinemia: Update on Epidemiology and Therapy. J. Allergy Clin. Immunol. Pract..

[7] Boeren M., Meysman P., Laukens K., Ponsaerts P., Ogunjimi B., Delputte P. (2023). T cell immunity in HSV-1- and VZV-infected neural ganglia. Trends Microbiol..

[8] Phares T.W., Stohlman S.A., Hwang M., Min B., Hinton D.R., Bergmann C.C. (2012). CD4 T cells promote CD8 T cell immunity at the priming and effector site during viral encephalitis. J. Virol..

[9] Lauver M.D., Jin G., Ayers K.N., Carey S.N., Specht C.S., Abendroth C.S., Lukacher A.E. (2022). T cell deficiency precipitates antibody evasion and emergence of neurovirulent polyomavirus. eLife.

[10] Krstanović F., Mihalić A., Rashidi A.S., Sitnik K.M., Ruzsics Z., Čičin-Šain L., Verjans G.M.G.M., Jonjić S., Brizić I. (2025). Neuron-restricted cytomegalovirus latency in the central nervous system regulated by CD4+ T-cells and IFN-γ. J. Neuroinflamm..

[11] Bergmann C.C., Ramakrishna C., Kornacki M., Stohlman S.A. (2001). Impaired T cell immunity in B cell-deficient mice following viral central nervous system infection. J. Immunol..

[12] National Institutes of Health, HIV Medicine Association, Infectious Diseases Society of America (2026). Panel on Guidelines for the Prevention and Treatment of Opportunistic Infections in Adults and Adolescents with HIV. Guidelines for the Prevention and Treatment of Opportunistic Infections in Adults and Adolescents with HIV.

[13] Schmidt-Hieber M., Schwender J., Heinz W.J., Zabelina T., Kühl J.S., Mousset S., Schüttrumpf S., Junghanss C., Silling G., Basara N. (2011). Viral encephalitis after allogeneic stem cell transplantation: A rare complication with distinct characteristics of different causative agents. Haematologica.

[14] Toomey D., Phan T.L., Phan T., Hill J.A., Zerr D.M. (2023). Viral Encephalitis after Hematopoietic Cell Transplantation: A Systematic Review. Transplant. Cell. Ther..

[15] Wollebo H.S., White M.K., Gordon J., Berger J.R., Khalili K. (2015). Persistence and pathogenesis of the neurotropic polyomavirus JC. Ann. Neurol..

[16] Tan I.L., Smith B.R., Von Geldern G., Mateen F.J., McArthur J.C. (2012). HIV-associated opportunistic infections of the CNS. Lancet Neurol..

[17] Bellier L.M., Kaminski H., Merville P., Couzi L. (2026). Interactions Between Immunosuppressive Regimens and Cytomegalovirus Infection After Solid-Organ Transplantation. Transpl. Int. Off. J. Eur. Soc. Organ Transplant..

[18] Tan C.S., Koralnik I.J. (2010). Progressive multifocal leukoencephalopathy and other disorders caused by JC virus: Clinical features and pathogenesis. Lancet Neurol..

[19] Soresina A., Galli J., Bellicini I., Gambara S., Scaduto R., Roversi S., Tozzo A., Nardocci N., Pinelli L., Hincks J. (2025). Pocapavir treatment of enterovirus encephalitis in a patient with X-linked Agammaglobulinemia. Clin. Immunol..

[20] Jones T.P., Buckland M., Breuer J., Lowe D.M. (2019). Viral Infection in Primary Antibody Deficiency Syndromes. Rev. Med. Virol..

[21] Chetty K., Cheng I., Kaliakatsos M., Gonzalez-Granado L.I., Klapsa D., Martin J., Bamford A., Breuer J., Booth C. (2022). Case Report: Novel Treatment Regimen for Enterovirus Encephalitis in SCID. Front. Immunol..

[22] Verma A.K., Perlman S. (2025). Unraveling the complexities of neurotropic virus infection and immune evasion. Microbiol. Mol. Biol. Rev. MMBR.

[23] Whitley R.J., Gnann J.W. (2002). Viral encephalitis: Familiar infections and emerging pathogens. Lancet.

[24] Boardman S.A., Hetherington C., Hughes T., Cook C., Galea I., Hilton O., Solomon T., Luster A.D., Allan S., Kurt-Jones E. (2025). Viral Infection and the Blood-Brain Barrier: Molecular Research Insights and Therapies. J. Infect. Dis..

[25] Harberts E., Yao K., Wohler J.E., Maric D., Ohayon J., Henkin R., Jacobson S. (2011). Human herpesvirus-6 entry into the central nervous system through the olfactory pathway. Proc. Natl. Acad. Sci. USA.

[26] Zubair A.S., McAlpine L.S., Gardin T., Farhadian S., Kuruvilla D.E., Spudich S. (2020). Neuropathogenesis and Neurologic Manifestations of the Coronaviruses in the Age of Coronavirus Disease 2019: A Review. JAMA Neurol..

[27] Tyler K.L. (2018). Acute Viral Encephalitis. N. Engl. J. Med..

[28] Miller J.M., Binnicker M.J., Campbell S., Carroll K.C., Chapin K.C., Gonzalez M.D., Harrington A., Jerris R.C., Kehl S.C., Leal S.M. (2024). Guide to Utilization of the Microbiology Laboratory for Diagnosis of Infectious Diseases: 2024 Update by the Infectious Diseases Society of America (IDSA) and the American Society for Microbiology (ASM). Clin. Infect. Dis..

[29] Debiasi R.L., Tyler K.L. (2004). Molecular methods for diagnosis of viral encephalitis. Clin. Microbiol. Rev..

[30] Shoskes A., Hassett C., Dani D., Majeed A. (2022). Pearls & Oy-Sters: Seronegative Eastern Equine Encephalitis in an Immunocompromised Stem Cell Transplant Recipient. Neurology.

[31] Wilson M.R., Sample H.A., Zorn K.C., Arevalo S., Yu G., Neuhaus J., Federman S., Stryke D., Briggs B., Langelier C. (2019). Clinical Metagenomic Sequencing for Diagnosis of Meningitis and Encephalitis. N. Engl. J. Med..

[32] Sonneville R., Magalhaes E., Meyfroidt G. (2017). Central nervous system infections in immunocompromised patients. Curr. Opin. Crit. Care.

[33] Chen J.-H.M., Wu J., Yang X.-Y.M., Li J.M., Huang N.-Q.M., Shi S.-P.M., Feng F.M., Li Q.B., Yu C.-Y., Luo Y. (2022). Diagnostic Value of the Electroencephalogram and Cerebrospinal Fluid in Viral Encephalitis. Neurol..

[34] Sutter R., Kaplan P.W., Cervenka M.C., Thakur K.T., Asemota A.O., Venkatesan A., Geocadin R.G. (2015). Electroencephalography for diagnosis and prognosis of acute encephalitis. Clin. Neurophysiol. Off. J. Int. Fed. Clin. Neurophysiol..

[35] Viarasilpa T., Panyavachiraporn N., Osman G., Parres C., Varelas P., Van Harn M., Mayer S.A. (2019). Electrographic Seizures in Patients with Acute Encephalitis. Neurocrit. Care.

[36] Iacoangeli M., Roselli R., Antinori A., Ammassari A., Murri R., Pompucci A., Scerrati M. (1994). Experience with brain biopsy in acquired immune deficiency syndrome-related focal lesions of the central nervous system. Br. J. Surg..

[37] Su L.D., Chiu C.Y., Gaston D., Hogan C.A., Miller S., Simon D.W., Thakur K.T., Yang S., Piantadosi A. (2024). Clinical Metagenomic Next-Generation Sequencing for Diagnosis of Central Nervous System Infections: Advances and Challenges. Mol. Diagn. Ther..

[38] Tang W., Gai Q., Yang J., Chen J., Lyu Z. (2026). PhIP-Seq: Unveiling the complexity of antibody repertoires in health and disease. Front. Immunol..

[39] Kostyusheva A., Brezgin S., Babin Y., Vasilyeva I., Glebe D., Kostyushev D., Chulanov V. (2022). CRISPR-Cas systems for diagnosing infectious diseases. Methods.

[40] You D., Xu T., Huang B.-Z., Zhu L., Wu F., Deng L.-S., Liu Z.-Y., Duan J.-Q., Wang Y.-M., Ge L.-P. (2024). Rapid, sensitive, and visual detection of swine Japanese encephalitis virus with a one-pot RPA-CRISPR/EsCas13d-based dual readout portable platform. Int. J. Biol. Macromol..

[41] Fortuna D., Hooper D.C., Roberts A.L., Harshyne L.A., Nagurney M., Curtis M.T. (2018). Potential role of CSF cytokine profiles in discriminating infectious from non-infectious CNS disorders. PLoS ONE.

[42] Handley G., Pankow S., Bard J.D., Yee R., Nigo M., Hasbun R. (2021). Distinguishing cytomegalovirus meningoencephalitis from other viral central nervous system infections. J. Clin. Virol. Off. Publ. Pan Am. Soc. Clin. Virol..

[43] Noguchi T., Yoshiura T., Hiwatashi A., Togao O., Yamashita K., Nagao E., Uchino A., Hasuo K., Atsumi K., Matsuura T. (2010). CT and MRI findings of human herpesvirus 6-associated encephalopathy: Comparison with findings of herpes simplex virus encephalitis. AJR Am. J. Roentgenol..

[44] Venkatesan A., Michael B.D., Probasco J.C., Geocadin R.G., Solomon T. (2019). Acute encephalitis in immunocompetent adults. Lancet.

[45] Liu Z., Peng A., Huang L., Sha L., Tang Y., Zhou Y., Chen L. (2025). Clinical features and risk factors for Epstein-Barr virus-associated encephalitis: A retrospective cohort study. Virol. J..

[46] Srifuengfung G., Suppakitjanusant P., Chaisrimaneepan N. (2024). EBV-associated CNS infection in an immunocompetent adult: A case report and literature review. Clin. Case Rep..

[47] Lee G.-H., Kim J., Kim H.-W., Cho J.W. (2021). Clinical significance of Epstein-Barr virus in the cerebrospinal fluid of immunocompetent patients. Clin. Neurol. Neurosurg..

[48] Cheng H., Chen D., Peng X., Wu P., Jiang L., Hu Y. (2020). Clinical characteristics of Epstein-Barr virus infection in the pediatric nervous system. BMC Infect. Dis..

[49] Saylor D., Thakur K., Venkatesan A. (2015). Acute encephalitis in the immunocompromised individual. Curr. Opin. Infect. Dis..

[50] Ongrádi J., Ablashi D.V., Yoshikawa T., Stercz B., Ogata M. (2017). Roseolovirus-associated encephalitis in immunocompetent and immunocompromised individuals. J. Neurovirol..

[51] Leon L.L., de Lima R.G., Boffi L.C., Bindilatti R.N., Garlipp C.R., Costa S.C.B., Bonon S.H.A. (2021). Arbovirus, herpesvirus, and enterovirus associated with neurological syndromes in adult patients of a university hospital, 2017–2018. Rev. Soc. Bras. Med. Trop..

[52] Kampouri E., Handley G., Phan T.L., Lee Y.J., Shaw R., Carpenter P.A., Dadwal S.S., Chemaly R.F., Papanicolaou G.A., Ogata M. (2025). American Society for Transplantation and Cellular Therapy Series #9: Management of Human Herpesvirus 6B After Hematopoietic Cell Transplantation and Chimeric Antigen Receptor-T-Cell Therapy. Transplant. Cell. Ther..

[53] Corral Í., de la Maza S.S., Rodríguez M., Kawiorski M.-M., López-Martínez M.-J., Galán J.-C. (2018). Molecular detection of human herpesvirus 7 DNA in cerebrospinal fluid from adult patients with neurological disorders. J. Neurovirol..

[54] Mann I., Morado-Aramburo O., Hasbun R. (2024). Emerging shadows: HHV-8-associated encephalitis unveiled in a solid organ transplant recipient. Transpl. Infect. Dis. Off. J. Transplant. Soc..

[55] Berger J.R., Aksamit A.J., Clifford D.B., Davis L., Koralnik I.J., Sejvar J.J., Bartt R., Major E.O., Nath A. (2013). PML diagnostic criteria: Consensus statement from the AAN Neuroinfectious Disease Section. Neurology.

[56] Yamaguchi K., Yamamoto H., Izutsu K., Yuasa M., Kaji D., Nishida A., Ishiwata K., Takagi S., Yamamoto G., Asano-Mori Y. (2024). Fatal outcome of BK virus encephalitis in an allogeneic stem cell transplantation recipient. J. Infect. Chemother. Off. J. Jpn. Soc. Chemother..

[57] Jun J., Choi Y., Kim H., Lee S.H., Jeong J., Jung J. (2016). BK polyomavirus encephalitis in a patient with thrombotic microangiopathy after an allogeneic hematopoietic stem cell transplant. Transpl. Infect. Dis. Off. J. Transplant. Soc..

[58] Jones C. (2025). Human alpha-herpesvirus 1 (HSV-1) viral replication and reactivation from latency are expedited by the glucocorticoid receptor. J. Virol..

[59] Andreu S., Galdo-Torres D., Ripa I., Caballero O., Bello-Morales R., López-Guerrero J. (2025). From HSV-2 to HSV-1: A Change in the Epidemiology of Genital Herpes. J. Infect..

[60] Kolchinski A., Li M., Habis R., Bean P., Heck A.N., Probasco J.C., Hasbun R., Venkatesan A. (2025). Encephalitis in Immunocompromised vs Immunocompetent Patients: A Comparative Study. Open Forum Infect. Dis..

[61] Tan I.L., McArthur J.C., Venkatesan A., Nath A. (2012). Atypical manifestations and poor outcome of herpes simplex encephalitis in the immunocompromised. Neurology.

[62] Kennedy P.G.E. (2021). An overview of viral infections of the nervous system in the immunosuppressed. J. Neurol..

[63] Solomon T., Hooper C., Easton A., Rosala-Hallas A., Facer B., Moore P., Keller S.S., Whitfield T., Fernandez C., Kneen R. (2026). Safety and efficacy of adjunct dexamethasone in adults with herpes simplex virus encephalitis in the UK (DexEnceph): A multicentre, observer-blind, randomised, phase 3, controlled trial. Lancet Neurol..

[64] Lee D.H., Zuckerman R.A., AST Infectious Diseases Community of Practice (2019). Herpes simplex virus infections in solid organ transplantation: Guidelines from the American Society of Transplantation Infectious Diseases Community of Practice. Clin. Transplant..

[65] Beyar-Katz O., Bitterman R., Zuckerman T., Ofran Y., Yahav D., Paul M. (2020). Anti-herpesvirus prophylaxis, pre-emptive treatment or no treatment in adults undergoing allogeneic transplant for haematological disease: Systematic review and meta-analysis. Clin. Microbiol. Infect. Off. Publ. Eur. Soc. Clin. Microbiol. Infect. Dis..

[66] Heininger U., Seward J.F. (2006). Varicella. Lancet.

[67] Kawada J.-I. (2018). Neurological Disorders Associated with Human Alphaherpesviruses. Adv. Exp. Med. Biol..

[68] Herlin L.K., Hansen K.S., Bodilsen J., Larsen L., Brandt C., Andersen C.Ø., Hansen B.R., Lüttichau H.R., Helweg-Larsen J., Wiese L. (2021). Varicella Zoster Virus Encephalitis in Denmark from 2015 to 2019—A Nationwide Prospective Cohort Study. Clin. Infect. Dis..

[69] Pollak L., Dovrat S., Book M., Mendelson E., Weinberger M. (2012). Varicella zoster vs. herpes simplex meningoencephalitis in the PCR era. A single center study. J. Neurol. Sci..

[70] Wang J., Yuan Y., Zhang Y., Liu H., Han J., Yan Y. (2026). Varicella-zoster virus-associated central nervous system infection in immunocompromised vs. immunocompetent herpes zoster patients: A comparative study. Front. Immunol..

[71] Gilden D., Cohrs R.J., Mahalingam R., Nagel M.A. (2009). Varicella zoster virus vasculopathies: Diverse clinical manifestations, laboratory features, pathogenesis, and treatment. Lancet Neurol..

[72] Pergam S.A., Limaye A.P. (2009). Varicella Zoster Virus (VZV) in Solid Organ Transplant Recipients. Am. J. Transplant..

[73] Tomblyn M., Chiller T., Einsele H., Gress R., Sepkowitz K., Storek J., Wingard J.R., Young J.-A.H., Boeckh M.A. (2009). Guidelines for Preventing Infectious Complications among Hematopoietic Cell Transplantation Recipients: A Global Perspective. Biol. Blood Marrow Transplant..

[74] Lee R., Kim E.-J., Nho D., Cho S.-Y., Kwag D., Kim H.-J., Cho B.-S., Kim Y.-J., Park S., Lee S.-E. (2026). Real-World Effectiveness of Recombinant Zoster Vaccine in Allogeneic Hematopoietic Cell Transplant Recipients. Transplant. Cell. Ther..

[75] Chapuis A.G., Orozco J.J., Milano F. (2025). Intrathecal rituximab for the treatment of Epstein-Barr virus-associated encephalitis. Front. Immunol..

[76] Fujimoto H., Asaoka K., Imaizumi T., Ayabe M., Shoji H., Kaji M. (2003). Epstein-Barr virus infections of the central nervous system. Intern. Med..

[77] Law N., Logan C., Taplitz R. (2024). EBV Reactivation and Disease in Allogeneic Hematopoietic Stem Cell Transplant (HSCT) Recipients and Its Impact on HSCT Outcomes. Viruses.

[78] Gandhi M.K., Hoang T., Law S.C., Brosda S., O’rOurke K., Tobin J.W.D., Vari F., Murigneux V., Fink L., Gunawardana J. (2021). EBV-associated primary CNS lymphoma occurring after immunosuppression is a distinct immunobiological entity. Blood.

[79] Weinberg A., Li S., Palmer M., Tyler K.L. (2002). Quantitative CSF PCR in Epstein-Barr virus infections of the central nervous system. Ann. Neurol..

[80] Rafailidis P.I., Mavros M.N., Kapaskelis A., Falagas M.E. (2010). Antiviral treatment for severe EBV infections in apparently immunocompetent patients. J. Clin. Virol. Off. Publ. Pan Am. Soc. Clin. Virol..

[81] Prockop S., Doubrovina E., Suser S., Heller G., Barker J., Dahi P., Perales M.A., Papadopoulos E., Sauter C., Castro-Malaspina H. (2020). Off-the-shelf EBV-specific T cell immunotherapy for rituximab-refractory EBV-associated lymphoma following transplantation. J. Clin. Investig..

[82] Moghadamnia M., Delroba K., Heidari S., Rezaie Z., Dashti-Khavidaki S. (2025). Impact of antiviral prophylaxis on EBV viremia and posttransplant lymphoproliferative disorders in solid organ transplant recipients: A systematic review and meta-analysis. Virol. J..

[83] Lindsay J., Yong M.K., Greenwood M., Kong D.C.M., Chen S.C.A., Rawlinson W., Slavin M. (2020). Epstein-Barr virus related post-transplant lymphoproliferative disorder prevention strategies in allogeneic hematopoietic stem cell transplantation. Rev. Med. Virol..

[84] Münz C. (2025). Epstein-Barr virus pathogenesis and emerging control strategies. Nat. Rev. Microbiol..

[85] Ljungman P., Alain S., Chemaly R.F., Einsele H., Galaverna F., Hirsch H.H., Sadowska-Klasa A., Navarro D., Styczynski J., de la Camara R. (2025). Recommendations from the 10th European Conference on Infections in Leukaemia for the management of cytomegalovirus in patients after allogeneic haematopoietic cell transplantation and other T-cell-engaging therapies. Lancet Infect. Dis..

[86] Ljungman P., de la Camara R., Robin C., Crocchiolo R., Einsele H., Hill J.A., Hubacek P., Navarro D., Cordonnier C., Ward K.N. (2019). Guidelines for the management of cytomegalovirus infection in patients with haematological malignancies and after stem cell transplantation from the 2017 European Conference on Infections in Leukaemia (ECIL 7). Lancet Infect. Dis..

[87] Toomey D., Phan T.L., Nguyen V., Phan T.T., Ogata M. (2021). Retrospective case analysis of antiviral therapies for HHV-6 encephalitis after hematopoietic stem cell transplantation. Transpl. Infect. Dis..

[88] Bhanushali M.J., Kranick S.M., Freeman A.F., Cuellar-Rodriguez J.M., Battiwalla M., Gea-Banacloche J.C., Hickstein D.D., Pavletic S., Fahle G., Nath A. (2013). Human herpes 6 virus encephalitis complicating allogeneic hematopoietic stem cell transplantation. Neurology.

[89] Razonable R.R. (2013). Human herpesviruses 6, 7 and 8 in solid organ transplant recipients. Am. J. Transplant. Off. J. Am. Soc. Transplant. Am. Soc. Transpl. Surg..

[90] Pellett Madan R., Hand J., AST Infectious Diseases Community of Practice (2019). Human herpesvirus 6, 7, and 8 in solid organ transplantation: Guidelines from the American Society of Transplantation Infectious Diseases Community of Practice. Clin. Transplant..

[91] Cortese I., Reich D.S., Nath A. (2021). Progressive multifocal leukoencephalopathy and the spectrum of JC virus-related disease. Nat. Rev. Neurol..

[92] Lindå H., von Heijne A., Major E.O., Ryschkewitsch C., Berg J., Olsson T., Martin C. (2009). Progressive Multifocal Leukoencephalopathy after Natalizumab Monotherapy. N. Engl. J. Med..

[93] Tan K., Roda R., Ostrow L., McArthur J., Nath A. (2009). PML-IRIS in patients with HIV infection: Clinical manifestations and treatment with steroids. Neurology.

[94] Fournier A., Martin-Blondel G., Lechapt-Zalcman E., Dina J., Kazemi A., Verdon R., Mortier E., de La Blanchardière A. (2017). Immune Reconstitution Inflammatory Syndrome Unmasking or Worsening AIDS-Related Progressive Multifocal Leukoencephalopathy: A Literature Review. Front. Immunol..

[95] Mendoza M.A., Imlay H. (2025). Polyomaviruses After Allogeneic Hematopoietic Stem Cell Transplantation. Viruses.

[96] Hirsch H.H., Randhawa P.S., AST Infectious Diseases Community of Practice (2019). BK polyomavirus in solid organ transplantation-Guidelines from the American Society of Transplantation Infectious Diseases Community of Practice. Clin. Transplant..

[97] Laskin B.L., Denburg M.R., Furth S.L., Moatz T., Altrich M., Kleiboeker S., Lutzko C., Zhu X., Blackard J.T., Jodele S. (2020). The Natural History of BK Polyomavirus and the Host Immune Response After Stem Cell Transplantation. Clin. Infect. Dis. Off. Publ. Infect. Dis. Soc. Am..

[98] (2024). Label: ACYCLOVIR Injection, Powder, Lyophilized, for Solution Food and Drug Administration. https://dailymed.nlm.nih.gov/dailymed/drugInfo.cfm?setid=babdbce2-5cbd-4943-bc38-9ebdd696a77a.

[99] Nau R., SörGel F., Eiffert H. (2010). Penetration of Drugs through the Blood-Cerebrospinal Fluid/Blood-Brain Barrier for Treatment of Central Nervous System Infections. Clin. Microbiol. Rev..

[100] (2025). Label: VALACYCLOVIR HYDROCHLORIDE Tablet, Film Coated Food and Drug Administration. https://dailymed.nlm.nih.gov/dailymed/drugInfo.cfm?setid=f5cfb61e-d971-4a9c-b9ef-4f3368fe02f5.

[101] (2026). Label: GANCICLOVIR-Ganciclovir Sodium Injection, Powder, Lyophilized, for Solution Food and Drug Administration. https://dailymed.nlm.nih.gov/dailymed/drugInfo.cfm?setid=b47f5d1c-36b8-49b6-a410-3b3f4661dde7#s27.

[102] (2026). Label: VALGANCICLOVIR HYDROCHLORIDE Tablet Food and Drug Administration. https://dailymed.nlm.nih.gov/dailymed/drugInfo.cfm?setid=8d2ffc05-2fbc-41f8-8656-fbc0bfa7ad83.

[103] (2025). Label: FOSCAVIR-Foscarnet Sodium Injection, Solution Food and Drug Administration. https://dailymed.nlm.nih.gov/dailymed/drugInfo.cfm?setid=90e3da4e-3b1f-428b-99ca-e4bed1c80028.

[104] (2021). Label: CIDOFOVIR-Cidofovir Anhydrous Injection, Solution Food and Drug Administration. https://dailymed.nlm.nih.gov/dailymed/drugInfo.cfm?setid=56541229-8c1a-4550-8951-2415ed08e7e9.

[105] (2026). Label: LIVTENCITY-Maribavir Tablet, Coated Food and Drug Administration. https://dailymed.nlm.nih.gov/dailymed/drugInfo.cfm?setid=c94fc2c5-e840-4f18-b7d8-d5eacb26d3a0.

[106] (2024). Label: RIBAVIRIN Capsule Food and Drug Administration. https://dailymed.nlm.nih.gov/dailymed/drugInfo.cfm?setid=35f99f76-f2ef-4a81-91ff-285419664be3.

[107] Hosoya M., Mori S., Tomoda A., Mori K., Sawaishi Y., Kimura H., Shigeta S., Suzuki H. (2004). Pharmacokinetics and Effects of Ribavirin Following Intraventricular Administration for Treatment of Subacute Sclerosing Panencephalitis. Antimicrob. Agents Chemother..

[108] (2026). Label: VEKLURY-Remdesivir Injection Food and Drug Administration. https://dailymed.nlm.nih.gov/dailymed/drugInfo.cfm?setid=c0978fa8-53ff-4ca2-82a7-567fd3e958ca.

[109] Tempestilli M., Caputi P., Avataneo V., Notari S., Forini O., Scorzolini L., Marchioni L., Bartoli T.A., Castilletti C., Lalle E. (2020). Pharmacokinetics of Remdesivir and GS-441524 in Two Critically Ill Patients Who Recovered from COVID-19. J. Antimicrob. Chemother..

[110] (2025). Label: OSELTAMIVIR Capsule Food and Drug Administration. https://dailymed.nlm.nih.gov/dailymed/drugInfo.cfm?setid=82685c2f-791b-440f-a1c2-5a032da7adca#section-6.2.

[111] Jhee S.S., Yen M., Ereshefsky L., Leibowitz M., Schulte M., Kaeser B., Boak L., Patel A., Hoffmann G., Prinssen E.P. (2008). Low Penetration of Oseltamivir and Its Carboxylate into Cerebrospinal Fluid in Healthy Japanese and Caucasian Volunteers. Antimicrob. Agents Chemother..

[112] (2025). Label: RITUXAN-Rituximab Injection, Solution Food and Drug Administration. https://dailymed.nlm.nih.gov/dailymed/drugInfo.cfm?setid=b172773b-3905-4a1c-ad95-bab4b6126563.

[113] Bonthius D.J. (2023). Measles Virus and the Central Nervous System: An Update. Semin. Pediatr. Neurol..

[114] Patterson M.C. (2020). Neurological Complications of Measles (Rubeola). Curr. Neurol. Neurosci. Rep..

[115] Laksono B.M., de Vries R.D., McQuaid S., Duprex W.P., de Swart R.L. (2016). Measles Virus Host Invasion and Pathogenesis. Viruses.

[116] Gunasekaran P.K., Saini A.G. (2025). Subacute sclerosing panencephalitis. Semin. Pediatr. Neurol..

[117] Huiming Y., Chaomin W., Meng M., The Cochrane Collaboration (2005). Vitamin A for treating measles in children. Cochrane Database of Systematic Reviews.

[118] (2025). Measles. https://www.who.int/news-room/fact-sheets/detail/measles.

[119] Pal G. (2011). Effects of ribavirin on measles. J. Indian Med. Assoc..

[120] Roy Moulik N., Kumar A., Jain A., Jain P. (2013). Measles outbreak in a pediatric oncology unit and the role of ribavirin in prevention of complications and containment of the outbreak. Pediatr. Blood Cancer.

[121] Gebara A., Homeidat M., Keadan T., Fisher-Negev T., Oiknine-Djian E., Wolf D.G., Mechoulam H., Ekstein D., Amer R. (2025). Remdesivir Treatment Outcomes of Subacute Sclerosing Panencephalitis Presenting with Macular Necrotizing Retinitis: A Case Report. Ocul. Immunol. Inflamm..

[122] Acciani M., Zyla D., Niemeyer G., Harkins S., Parekh D., Pawlack E., Lacarbonara D., Kansara D., Ackerman M.E., Niewiesk S. (2026). Human neutralizing antibodies targeting the measles virus hemagglutinin and fusion surface proteins. Cell Host Microbe.

[123] Tahir I.M., Kumar V., Faisal H., Gill A., Kumari V., Tahir H.M., Haque A. (2024). Contagion comeback: Unravelling the measles outbreak across the USA. Front. Public Health.

[124] Garg R.K., Suresh V., Suvirya S., Rizvi I., Kumar N., Pandey S. (2024). Clinical features, pathogenesis, pathology, neuroimaging, clinical course and outcome of measles inclusion-body encephalitis: A systematic review of published case reports and case series. Neurol. Sci. Off. J. Ital. Neurol. Soc. Ital. Soc. Clin. Neurophysiol..

[125] Vidal L.R., de Almeida S.M., Cavalli B.M., Dieckmann T.G., Raboni S.M., Salvador G.L.O., Pereira L.A., Rotta I., Nogueira M.B. (2019). Human adenovirus meningoencephalitis: A 3-years’ overview. J. Neurovirol..

[126] Narsana N., Ha D., Ho D.Y. (2025). Treating Adenovirus Infection in Transplant Populations: Therapeutic Options Beyond Cidofovir?. Viruses.

[127] Suresh S., Rawlinson W.D., Andrews P.I., Stelzer-Braid S. (2020). Global epidemiology of nonpolio enteroviruses causing severe neurological complications: A systematic review and meta-analysis. Rev. Med. Virol..

[128] Torres S.D., Jia D.T., Schorr E.M., Park B.L., Boubour A., Boehme A., Ankam J.V., Gofshteyn J.S., Tyshkov C., Green D.A. (2020). Central nervous system (CNS) enterovirus infections: A single center retrospective study on clinical features, diagnostic studies, and outcome. J. Neurovirol..

[129] Wagner J.N., Leibetseder A., Troescher A., Panholzer J., von Oertzen T.J. (2021). Characteristics and therapy of enteroviral encephalitis: Case report and systematic literature review. Int. J. Infect. Dis. IJID Off. Publ. Int. Soc. Infect. Dis..

[130] Dixon L., McNamara C., Dhasmana D., Taylor G.P., Davies N. (2023). Imaging Spectrum of HTLV-1–Related Neurologic Disease: A Pooled Series and Review. Neurol. Clin. Pract..

[131] Izumo S., Goto I., Itoyama Y., Okajima T., Watanabe S., Kuroda Y., Araki S., Mori M., Nagataki M., Matsukura S. (1996). Interferon-alpha is effective in HTLV-I-associated myelopathy: A multicenter, randomized, double-blind, controlled trial. Neurology.

[132] Tsutsumi S., Sato T., Yagishita N., Yamauchi J., Araya N., Hasegawa D., Nagasaka M., Coler-Reilly A.L.G., Inoue E., Takata A. (2019). Real-world clinical course of HTLV-1-associated myelopathy/tropical spastic paraparesis (HAM/TSP) in Japan. Orphanet J. Rare Dis..

[133] Sato T., Coler-Reilly A.L.G., Yagishita N., Araya N., Inoue E., Furuta R., Watanabe T., Uchimaru K., Matsuoka M., Matsumoto N. (2018). Mogamulizumab (Anti-CCR4) in HTLV-1–Associated Myelopathy. N. Engl. J. Med..

[134] Wei J., Huang H., Wu X., Xu Y., Wang X. (2026). Pathogenesis and Research Models of Acute Influenza-Associated Encephalitis/Encephalopathy: An Update. Viruses.

[135] Silverman A., Walsh R., Santoro J.D., Thomas K., Ballinger E., Fisher K.S., Thomas A.X., Appavu B., Kruer M.C., Influenza-Associated Acute Necrotizing Encephalopathy (IA-ANE) Working Group (2025). Influenza-Associated Acute Necrotizing Encephalopathy in US Children. JAMA.

[136] Sayyad L.E., Smith K.L., Sadigh K.S., Cossaboom C.M., Choi M.J., Whitmer S., Cannon D., Krapiunaya I., Morales-Betoulle M., Annambhotla P. (2025). Severe Non-Donor-Derived Lymphocytic Choriomeningitis Virus Infection in 2 Solid Organ Transplant Recipients. Open Forum Infect. Dis..

[137] Anesi J.A., Silveira F.P., AST Infectious Diseases Community of Practice (2019). Arenaviruses and West Nile Virus in solid organ transplant recipients: Guidelines from the American Society of Transplantation Infectious Diseases Community of Practice. Clin. Transplant..

[138] Zamani R., Pouremamali R., Rezaei N. (2022). Central neuroinflammation in COVID-19: A systematic review of 182 cases with encephalitis, acute disseminated encephalomyelitis, and necrotizing encephalopathies. Rev. Neurosci..

[139] Ferreira M.L.B., Albuquerque M.d.F.P.M.d., de Brito C.A.A., França R.F.d.O., Moreira Á.J.P., Machado M.Í.d.M., Melo R.d.P., Medialdea-Carrera R., Mesquita S.D., Santos M.L. (2020). Neurological Disease in Adults with Zika and Chikungunya Virus Infection in Northeast Brazil: A Prospective Observational Study. Lancet. Neurol..

[140] Staples J.E., Gould C.V. (2025). Eastern Equine Encephalitis in the US. JAMA.

[141] Coates E.E., Edupuganti S., Chen G.L., Happe M., Strom L., Widge A., Florez M.B., Cox J.H., Gordon I., Plummer S. (2022). Safety and Immunogenicity of a Trivalent Virus-like Particle Vaccine against Western, Eastern, and Venezuelan Equine Encephalitis Viruses: A Phase 1, Open-Label, Dose-Escalation, Randomised Clinical Trial. Lancet Infect. Dis..

[142] Wang L., Zheng R., Li Z., Zhang L. (2025). Western Equine Encephalitis Virus: A Comprehensive Review of Epidemics, Transmission, Hosts, and Strategies for Mitigation. Virulence.

[143] Bowen G.S., Fashinell T.R., Dean P.B., Gregg M.B. (1976). Clinical Aspects of Human Venezuelan Equine Encephalitis in Texas. Bull. Pan Am. Health Organ..

[144] Meier-Stephenson V., Drebot M.A., Dimitrova K., DiQuinzio M., Fonseca K., Forrest D., Hatchette T., Morshed M., Patriquin G., Poliquin G. (2024). Case Series of Jamestown Canyon Virus Infections with Neurologic Outcomes, Canada, 2011-2016. Emerg. Infect. Dis..

[145] McJunkin J.E., Reyes E.C.d.L., Irazuzta J.E., Caceres M.J., Khan R.R., Minnich L.L., Fu K.D., Lovett G.D., Tsai T., Thompson A. (2001). La Crosse Encephalitis in Children. N. Engl. J. Med..

[146] Ho B.M., Davis H.E., Forrester J.D., Sheele J.M., Haston T., Sanders L., Lee M.C., Lareau S., Caudell M., Davis C.B. (2021). Wilderness Medical Society Clinical Practice Guidelines for the Prevention and Management of Tick-Borne Illness in the United States. Wilderness Environ. Med..

[147] Colorado Tick Fever (CTF) (2022). Tickborne Diseases of the United States: A Reference Manual for Healthcare Providers.

[148] Noronha A.K., Rupali P. (2025). Dengue Encephalitis: What’s New?. Curr. Opin. Infect. Dis..

[149] Guo H., Sun L., Shen X., Hu W. (2022). A Retrospective Study of the Clinical Characteristics of Japanese Encephalitis in Adults. J. Integr. Neurosci..

[150] Lin F.-H., Chou Y.-C., Hsieh C.-J., Yu C.-P. (2025). Epidemiological Features, Clinical Symptoms, and Environmental Risk Factors for Notifiable Japanese Encephalitis in Taiwan from 2008 to 2020: Retrospective Study. JMIR Public Health Surveill..

[151] Solomon T. (2004). Flavivirus Encephalitis. N. Engl. J. Med..

[152] Mendoza M.A., Hass R.M., Vaillant J., Johnson D.R., Theel E.S., Toledano M., Abu Saleh O. (2024). Powassan Virus Encephalitis: A Tertiary Center Experience. Clin. Infect. Dis. Off. Publ. Infect. Dis. Soc. Am..

[153] Solomon I.H., Spera K.M., Ryan S.L., Helgager J., Andrici J., Zaki S.R., Vaitkevicius H., Leon K.E., Wilson M.R., DeRisi J.L. (2018). Fatal Powassan Encephalitis (Deer Tick Virus, Lineage II) in a Patient With Fever and Orchitis Receiving Rituximab. JAMA Neurol..

[154] Pedrosa D.A., Oliveira L.K.L.d.P., Bertanha R., Júnior E.A., Fernandes G.B.P., Thomaz R.B. (2024). Acute Disseminated Encephalomyelitis Following Saint Louis Encephalitis Virus Infection. Neurol. Sci. Off. J. Ital. Neurol. Soc. Ital. Soc. Clin. Neurophysiol..

[155] Curren E.J., Lindsey N.P., Fischer M., Hills S.L. (2018). St. Louis Encephalitis Virus Disease in the United States, 2003–2017. Am. J. Trop. Med. Hyg..

[156] Hills S.L., Poehling K.A., Chen W.H., Staples J.E. (2023). Tick-Borne Encephalitis Vaccine: Recommendations of the Advisory Committee on Immunization Practices, United States, 2023. MMWR Recomm. Rep. Morb. Mortal. Wkly. Rep. Recomm. Rep..

[157] Chiffi G., Grandgirard D., Leib S.L., Chrdle A., Růžek D. (2023). Tick-borne Encephalitis: A Comprehensive Review of the Epidemiology, Virology, and Clinical Picture. Rev. Med. Virol..

[158] Gould C.V., Staples J.E., Guagliardo S.A.J., Martin S.W., Lyons S., Hills S.L., Nett R.J., Petersen L.R. (2025). West Nile Virus: A Review. JAMA.

